# Biochemical and Molecular Dynamic Simulation Analysis of a Weak Coiled Coil Association between Kinesin-II Stalks

**DOI:** 10.1371/journal.pone.0045981

**Published:** 2012-09-28

**Authors:** Harinath Doodhi, Swadhin C. Jana, Pavithra Devan, Shyamalava Mazumdar, Krishanu Ray

**Affiliations:** 1 Department of Biological Sciences, Tata Institute of Fundamental Research, Mumbai, India; 2 Department of Chemical Sciences, Tata Institute of Fundamental Research, Mumbai, India; Russian Academy of Sciences, Institute for Biological Instrumentation, Russian Federation

## Abstract

**Definition:**

Kinesin-2 refers to the family of motor proteins represented by conserved, heterotrimeric kinesin-II and homodimeric Osm3/Kif17 class of motors.

**Background:**

Kinesin-II, a microtubule-based anterograde motor, is composed of three different conserved subunits, named KLP64D, KLP68D and DmKAP in *Drosophila*. Although previous reports indicated that coiled coil interaction between the middle segments of two dissimilar motor subunits established the heterodimer, the molecular basis of the association is still unknown.

**Methodology/Principal Findings:**

Here, we present a detailed heterodimeric association model of the KLP64D/68D stalk supported by extensive experimental analysis and molecular dynamic simulations. We find that KLP64D stalk is unstable, but forms a weak coiled coil heteroduplex with the KLP68D stalk when coexpressed in bacteria. Local instabilities, relative affinities between the C-terminal stalk segments, and dynamic long-range interactions along the stalks specify the heterodimerization. Thermal unfolding studies and independent simulations further suggest that interactions between the C-terminal stalk fragments are comparatively stable, whereas the N-terminal stalk reversibly unfolds at ambient temperature.

**Conclusions/Significance:**

Results obtained in this study suggest that coiled coil interaction between the C-terminal stalks of kinesin-II motor subunits is held together through a few hydrophobic and charged interactions. The N-terminal stalk segments are flexible and could uncoil reversibly during a motor walk. This supports the requirement for a flexible coiled coil association between the motor subunits, and its role in motor function needs to be elucidated.

## Introduction

Kinesin-II is a conserved microtubule-based motor implicated in the anterograde intraflagellar transport (IFT) [1], [2], [3], cilia assembly [4], axonal transport [5], [6], and many other intracellular processes. It consists of two different motor (A and B) subunits and a nonmotor accessory subunit (KAP). Genetic analysis indicated that it functions as a heterotrimer *in vivo* [7], [8], [9], [10], [11]. Previous studies also showed that the kinesin-II motor subunits associate with each other *in vitro* independent of KAP [6], [12], which binds to the coiled-coil stalks of the motor subunits [13]. In addition, kinesin-II motors klp11 and klp20 of *C. elegans* become processive upon heterodimerization with each other [14]. Together, these data suggest that association between the kinesin-II motor subunits is essential for the holoenzyme assembly and biochemical functioning of the motor *in vivo*.

A majority of the kinesin like proteins homodimerize through two stranded coiled coil association. Kinesin-II motor subunits contain three predicted coiled coil domains in the middle, and always form a heterodimer [14], [15], [16], [17]. Earlier studies suggested that the complementary charges on the amino acid residues in the respective hinge regions would prevent homodimerization between the mouse kinesin-II motor subunits [17], [18]. Comprehensive and rigorous analysis using the *Xenopus* homologues, Xklp3A and 3B, however, indicated that the C-terminal parts of the individual stalks determine the process [16], [19]. Recently, the minimal and essential region for dimerization is further mapped to two heptads at the C-terminal end of the stalks of the *C. elegans* kinesin-II [20]. Unlike many other coiled coil polypeptides [21], [22], [23], kinesin-II stalk segments lack canonical *trigger* sequences and contain several atypical heptads with charged amino acid residues in the ‘*a*’ and ‘*d*’ positions, which might results in either underwinding or overwinding of the coiled coil [24]. The C-terminal halves of Xklp3A and 3B (of *X. laevis*) [19], and KLP11 and KLP20 (of *C. elegans*) [20] formed a coiled coil heterodimer. Further it was observed that the stability and alpha helicity of the C-terminal and full length stalks of KLP11 and KLP20 are comparable, implying that N-terminal half does not form a coiled coil. However, FRET analysis of the full length stalk indicates the entire stalks forms a heterodimer [20]. Thus, assembly of the kinesin-II stalk provides an intriguing exception to the canonical coiled coil theory. However, in spite of obtaining diffracting crystals of recombinant kinesin-II stalk [19] an atomic resolution model is still unavailable.

**Figure 1:**
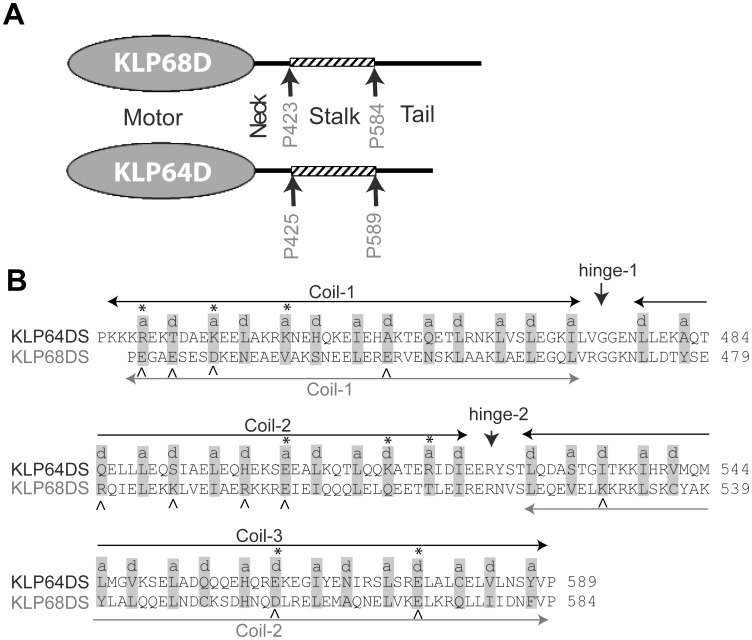
Stalk region of *Drosophila* Kinesin-II motor subunits has the propensity to form coiled coil. A) Schematic illustrates domain organizations in the KLP64D and KLP68D and highlight the predicted ‘stalk’ fragments between P425-P589 in KLP64D and the P423–P584 in KLP68D. B) Coiled-coil forming regions in the KLP64D and KLP68D stalks as predicted by the MultiCoil program [33]. The coiled-coil domains (shown by arrows) and the heptad-repeat assignment (*a* and *d* positions of the heptad-repeat (*a-b-c-d-e-f-g*) in grey background)for KLP64DS and KLP68DS based on MultiCoil program and visual inspection are shown above the amino acid sequence. The charged amino acids in the ‘*a*’ and ‘*d*’ positons are marked with ‘*’ and ‘^∧^’ on KLP64D and KLP68D respectively.

Computer simulation provides a powerful alternative to gain insight into such, difficult to obtain, protein structures [25], [26]. Interactions between KAP and the motor subunits [13], and dynamic properties of coiled coils [27], [28], are some examples where such an approach, combined with experimental verifications, elicited novel insights. Here, we report an analysis of interactions between isolated stalk fragments of *Drosophila* kinesin-II motor subunits, KLP64D (A-subunit) and KLP68D (B-subunit) (Figure 1A), using Circular dichroism and thermal unfolding analysis of the full length and N-terminal stalks, and molecular dynamic (MD) simulations. Together, the data presented here suggest that complementary instability and affinity between the opposite stalk segments drives heterodimerization, possibly through cooperative folding. We also found that the C-terminal stalk segments are necessary to initiate the process, which is similar to the Xklp3A/B [16] and KLP11/20 [20] stalks. Further, we report the instability and the lower alpha helicity of the purified N-terminal stalks. Additionally, MD simulations show that charge interactions along the lengths of the stalk fragments stabilize the heteroduplex. The results also indicate that the segments proximal to the motor domain (N-terminal stalk) are flexible and reversibly unfold at room temperature. Thus, we propose that the N-terminal portion of kinesin-II stalk may uncoil during motor walk, which might contribute to the reportedly low processivity of the motor.

## Results

Kinesin stalks form through coiled coil interactions (*reviewed in* [29], [30]. The secondary structure predictions using PSIPRED [31], [32] indicated that most of the middle portions of both the KLP64D (P425–P589) and KLP68D (P423–P584) would be alpha-helical (Figure S1), and the MultiCoil program [33] predicted that they would form coiled coils. We denoted this segment as the stalk (Figure 1A). The KLP64D stalk (KLP64DS) is predicted to contain three coiled coils (Coils 1, 2 and 3) interspersed with two short breaks, whereas, KLP68D stalk contains only two coils with a large gap in the middle. (Figure S2 and Figure 1B). The coiled coil sequences contain a characteristic seven residue periodicity (heptad repeat) denoted as ‘*a-b-c-d-e-f-g*’. The ‘a’ and ‘*d*’ positions are predominantly occupied by hydrophobic amino acids. Whereas charged amino acids dominate in the ‘*e*’ and ‘*g*’ positions (*reviewed in* [34], [35]. However, MultiCoil prediction and manual inspection of the amino acid sequences of KLP64DS and KLP68DS revealed frequent occurrences of charged amino acid residues in the '*a*' and '*d*' positions (Figure 1B). Unlike the kinesin heavy chain [22], [36] and other vertebrate homologues of kinesin-II motor subunits [17], [18], the neck sequences (between the motor domain and the stalk) of KLP64D and KLP68D have remarkably little or no coiled coil forming propensity (Figure S2).

### KLP64D Stalk Misfolds into an Insoluble form in the Absence of the KLP68D Stalk

**Figure 2:**
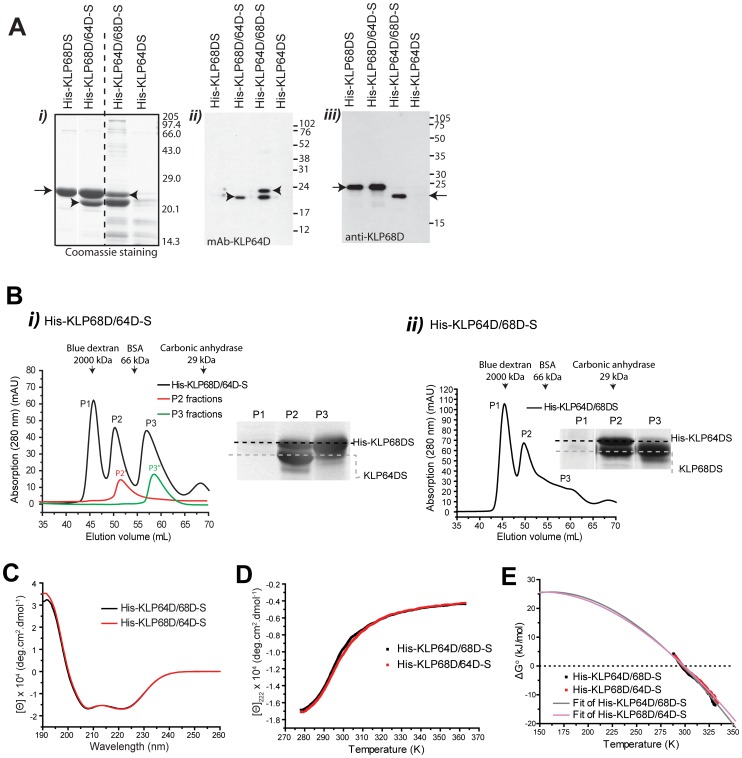
KLP64D and KLP68D stalks heterodimerize through coiled-coil interaction. A) Expression and affinity purification of the kinesin-II stalk fragments. *i)* Coomasssie stained gel shows affinity purified products of the individual and combinatorial expression. Arrowheads indicate the KLP64DS bands and the arrow indicates the His-KLP68DS. *ii)* mAb-KLP64D staining of the western blot of a gel similar to the one presented in (*i*) show that recombinant His-KLP64DS (lane 3) migrates in two distinct forms in SDS-PAGE. *iii)* Anti-KLP68D staining of a similar blot revealed the corresponding KLP68DS band (arrows). B) Gel filtration analysis of the affinity purified His-KLP68D/64D-S heterodimer using HiLoad 16/60 Superdex™ 75 pg column: *i*) fraction-wise absorption profiles of the affinity purified protein (black line), the pooled P2 (red), and the P3 (green) fractions. Coomassie staining of the SDS-PAGE gels of representative fractions from the P1, P2 and P3 sets. Labeled dashed lines mark the position of respective bands on a coomassie stained gels. *ii*) Gel filtration analysis of His-KLP64D/68D-S indicates that the heterodimer elutes in the P2 range. C) Mean residue molar ellipticity ([Θ]) values of the purified His-KLP64D/68D-S (black curve) and His-KLP68D/64D-S (red curve) heterodimers measured in the far-UV wavelengths by using a circular dichroism (CD) spectrometer at 5°C (278K). D) Plot of [Θ]_222_ values of His-KLP64D/68D-S and H-KLP68D/64D-S at different temperatures. E) Stability curves of His-KLP64D/68D-S (black squares) and His-KLP68D/64D-S (red squares), each point in the stability curve represents the free energy of unfolding (ΔG) at corresponding temperature. The solid line represents the non-linear curve fits to Gibbs-Helmholtz equation (Eq. 6).

The C-terminal stalk fragments of Xklp3A and 3B formed a stable heterodimer when they were coexpressed in *E. coli* [19]. Therefore, the KLP64DS and KLP68DS were cloned in pETDuet-1 for separate and combined expressions (Table S1). Affinity purification (Figure 2A-*i*) and subsequent immunostaining (Figure 2A-*ii* and *iii*) of the western blots revealed that His-KLP68DS was soluble (arrow, Figure 2A), and KLP64DS copurified with the His-KLP68DS when they were coexpressed (arrowhead, Figure 2A). This suggested that KLP64DS needs KLP68DS to fold together into a soluble heterodimer. We noticed that His-KLP64DS, copurified with KLP68DS, always migrated in two forms in the SDS-PAGE. This is indicated by the presence of two closely spaced bands highlighted by the mAB-KLP64D immunostaining (lane 3, Figure 2A-*ii*). As a result, the lower band in Figure 2A-*i* (lane 3) stained more by the Coomassie blue. The affinity purified His-KLP68D/64D-S and His-KLP64D/68D-S heterodimers eluted in similar volume fractions upon gel filtration, (P2, Figure 2B). The excess His-KLP68DS eluted at a relatively smaller size fraction (P3, Figure 2B-*i*). We also found that both the band intensities are comparable for purified heterodimer (Figure 2B). Thus, the purified KLP64D and KLP68D stalk fragments is likely to heterodimerize in 1∶1 ratio. This is consistent with the compositions of vertebrate kinesin-II [12], [19], [37].

### KLP64D and KLP68D Stalks form a Weak Coiled-coil Heterodimer

Next, to verify the secondary structure of the purified polypeptides, we subjected them to Far-UV circular dichroism (CD) analysis, which is the appropriate secondary structure determination tool used for coiled-coil estimation [19], [38], [39]. The CD spectra of His-KLP68D/64D-S and His-KLP64D/68D-S heterodimers, collected in the P2 fractions, contained alpha-helical characteristic double minima at 208 and 222 nm (Figure 2C). A deconvolution analysis of the CD spectrum, using the Reed method [40] and reference standards provided by the manufacturer, indicated that both the samples contain mostly alpha-helical secondary structure (Table S2). However, analysis using the Yang method [41] and the one used by De Marco et al., (2003) [19] suggested <42% α-helix content (Table 1). The estimated [Θ]_222_/[Θ]_208_ values for purely alpha helical and coiled coils are 0.83 and 1.03, respectively [42], [43]. The [Θ]_222_/[Θ]_208_ ratios of KLP68D/64D-S are higher than those of a typical alpha-helix and somewhat closer to the coiled coil values (Table 1). We also noticed that addition of the His-tags at the N-terminus of the KLP68DS increases the yield and coiled-coil propensity of the heterodimer. Expectedly, [Θ]_222_/[Θ]_208_ ratios of His-KLP64D/68D-S decreased in the presence of 50% Trifluoroethanol (TFE) (Figure S3), known to promote coiled coil to alpha-helical transition in polypeptides. Together, these suggested that both types of the recombinant KLP64D/68D-S form a coiled-coil heteroduplex in solution. However, this raised an apparent contradiction since most coiled coils form between purely α-helical polypeptide chains.

**Table 1:** Circular Dichroism analysis of the full length, heterodimeric kinesin-II stalks.

Protein	No. of residues	[Θ]_222_ (deg.cm^2^.dmol^−1^)	α-helix content (%)	[Θ]_222_/[Θ]_208_ *	T_m_ (K)	Δ*H_m_*(kJ/mol)	*T_s_*_­_(K)	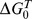 at 295K(kJ/mol)
His-KLP64D/68D-S	183+166	−16770.5	34.5%	0.996	297.6±0.1	102.1±1.5	161.7	0.90
His-KLP68D/64D-S	180+169	−16946.6	41.4%	1.024	298.8±0.1	94.9±0.8	153.4	1.19

The [Θ]_222_ is the ellipticity at 278K. Helical content is calculated by Yang’s method [41]. Tm is the Transition midpoint temperature; Δ*H_m_* is the enthalpy change at the midpoint of transition; *T_s_* is the temperature of maximum conformational stability of the protein; 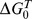 is the Gibbs free energy of unfolding.

* Typical value for a coiled coil is 1.03 and for an alpha-helix is 0.83 [42], [43].

To resolve this, we estimated the thermodynamic parameters, thermal stabilities of the heterodimers by measuring the [Θ] values between 5 and 90°C (278–363 K). The results were consistent at all wavelengths monitored. Therefore, we decided to analyze the melting profile at [Θ]_222_ which is most sensitive of changes in helical structure (Figure 2D), and used the transition range (∼288 to ∼334 K) denaturation profile for the T_m_ calculation. It suggested that both the His-KLP64D/68D-S (*T_m_* = 297.6 K) and His-KLP68D/64D-S (*T_m_* = 298.8 K) would unfold rapidly between 293 and 310 K (Figure 2D). The enthalpy change at the midpoint of transition, obtained by nonlinear least square fit of 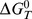
data to the Gibbs-Helmholtz equation (Eq. 6) (Figure 2E and Table 1) and the corresponding 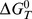
values and *T_S_* also appeared to be low (Table 1), indicating that the KLP64D/68D-S heterodimer is a weak coiled coil. These estimates are comparable to that of the N-terminal half of the NCD stalk, which exhibited reversible denaturation at low temperatures (20–30°C) [39]. Hence, the KLP64D/68D-S would be structurally unstable. In comparison, previous estimates indicated that the C-terminal halves of the Xklp3A/B stalk (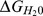 = 9.1 kcal/mol or 38.0 kJ/mol) [19] and the KLP11/20 stalk (*T_m_* = 39.14°C) [20] would be relatively more stable. The other coiled coils, *e.g.*, the kinesin heavy chain (khc) neck coiled coil, was reported to have a dissociation free energy of 57.7 kJ/mol [22], [23]. Further, the recombinant MyoVa stalk also exhibited high stability [43]. The amino acid sequences of the kinesin-II stalks from *Xenopus*, *C. elegans* and *Drosophila* are quite diverse (18% to 20% identity), which could account for the difference in the estimated functional characteristics.

The CD analysis, however, provides an estimate of the biophysical properties at thermodynamic equilibrium, which does not necessarily correspond to the mechanical properties of the stalk heterodimer. Indeed, single-molecule analysis showed that kinesin-1 neck coiled coil is mechanically stable, though it is thermodynamically less stable [44]. Furthermore, structural instability and dynamics of KLP64D/68-S could also cause low average absorbance in the corresponding CD spectra.

### Molecular Dynamic Simulation of a KLP64D/68D-S Model: an Incomplete Coiled-coil Stabilized by Distributed Interactions

**Figure 3:**
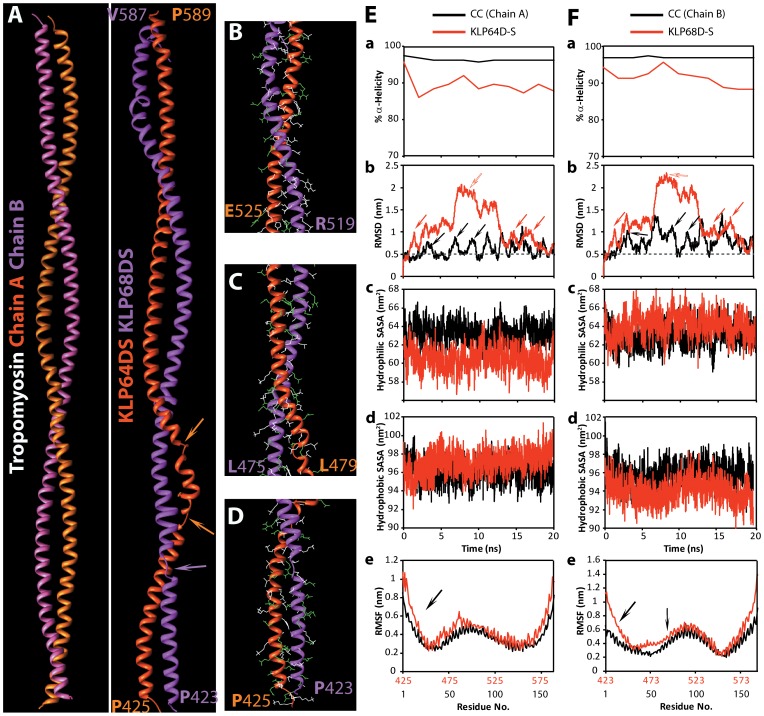
An atomic resolution model depicting stability and interactions between the KLP64D and KLP68D stalk polypeptides in a heterodimer. A) Ribbon structures of Tropomyosin Coiled-Coil homodimer (Chain A shown in dull orange and Chain B in hot pink) and KLP64D/68D-S heterodimer model (KLP64DS in orange red and KLP68DS in magenta) after 20 ns MD simulations at 300K. B–D) The backbone structures of the KLP64DS and KLP68DS the heterodimer model with the amino acid residues (blue and green) predicted to form atomic contacts in the C-terminal (B), middle (C) and N-terminal (D) regions. Amino acids with hydrophobic and charged side chains are marked in white and green, respectively (Table S4). E) Time evolution of % α-helicity (a), RMSD (b), hydrophilic-SASA (c), hydrophobic-SASA (d), and RMSF (Cα) as a function of residue number (e), respectively, of the tropomyosin (CC, chain A) and KLP64D at 300K (27°C). F) A similar time evolution of the same set of measurements as shown in E is also shown for a tropomyosin (CC, chainB) and KLP68D.

The secondary structure predictions and CD analysis suggested that the heterodimeric KLP64D/68D-S should form a coiled coil. Hence, we modelled its structure using the pig tropomyosin coiled coil as a template (PDB:1C1G) [13]. In order to understand the molecular characteristics of the coiled coil association between KLP64DS and KLP68DS, we simulated a coiled coil KLP64D/68D-S model under well defined boundary conditions by using standard molecular dynamic (MD) package [45] (for details see Materials and Methods). After 20 ns of MD simulations, 97% of a true Coiled-Coil (tropomyosin coiled coil, PDB:1C1G, called as CC henceforth) domain is alpha–helical. However, only 88% of the KLP64D/68D-S is estimated to retain alpha-helical structure after the simulation. This showed that the heterodimeric stalk model is less stable, and it could partly unfold at even 300 K (27°C) (Figures 3A, E and F). After 20 ns, the root mean square deviations (RMSD) of the backbone with respect to the corresponding starting structures indicated that a true coiled-coil only deviates up to 0.68 nm at 300 K. The RMSD of KLP64D/68D-S backbone was 0.68 nm at 300 K, which increased further to 2.4 nm at 373 K (100°C) (Table 2 and 3), suggesting that the KLP64D/68D-S heterodimer would unfold rapidly above 300 K. However, it was predicted to retain some residual structure even after a 20 ns simulation at 373 K. Both these predictions are consistent with the interpretations of the thermal denaturation data obtained by CD spectroscopy in this study (Figure 2D and E).

**Table 2:** Average RMSD, % α-Helicity, SASA and average number of different possible interactions in a pure Coiled-Coil (CC) and KLP64D/68D-S after 20 ns MD simulations at 300 K (averaged over last 100 ps).

	Coiled-Coil (CC)	KL64D/68D-S
RMSD (nm)	0.68	0.68
Rg (nm)	6.96	6.89
% α-helicity	96.7	88.2
SASA-Hydrophobic (nm^2^)	133	134.2
SASA-Hydrophilic (nm^2^)	115	106.8
No. of Hydrophobic interactions	98	44
No. of favourable Electrostatic interactions	225	210
No. of Salt Bridges	6	9

**Table 3:** Average RMSD, % α-helicity of KLP64D/68D-S after 20 ns MD simulations at different temperatures (averaged over last 100 ps).

	278 K	300 K	373 K
RMSD (nm)	0.69	0.68	2.4
% α-helicity	93	88.4	69.1
SASA-Hydrophobic (nm^2^)	134	134.2	134.4
SASA-Hydrophilic (nm^2^)	103.7	106.8	106.2

The simulations also revealed that the number of predicted hydrophobic interactions and favourable electrostatic interactions, assessed from the models at ambient temperature (300 K, 27°C), are considerably less in KLP64D/68D-S than those in the CC. While both the hydrophobic and hydrophilic amino acids are evenly distributed in the C-terminal part of the stalk, hydrophobic residues are less in the N-terminal region. Incidentally, the number of probable salt bridges predicted to be present in KLP64D/68D-S were little more than those found to remain in the CC after the 20 ns simulations (Table 2). These extra salt bridges perhaps compensate for the relatively less number of hydrophobic interactions and provide some stability to the heterodimer. In addition, the simulation also predicted that the hydrophobic- and hydrophilic- solvent accessible surface areas (SASA) of KLP64D/68D-S would be marginally higher as compared to the CC at 300 K (Figure 3, Table 2 and 3). A similar trend was observed in other three temperatures (data not shown). Although % α-helicity and SASA values of the KLP64D/68D-S model were altered significantly due to change in temperatures from 278 to 300 K during simulation, increasing the simulation temperature to 373 K caused additional significant deviations (Figure 3, Figures S4 and S5). This further implied that the weak coiled-coil structure could achieve an equilibrium solvent state at room temperature, which is also consistent with the CD spectroscopy of His-KLP64D/68D-S at different temperatures. This is discussed in further detail, in the following section.

**Figure 4:**
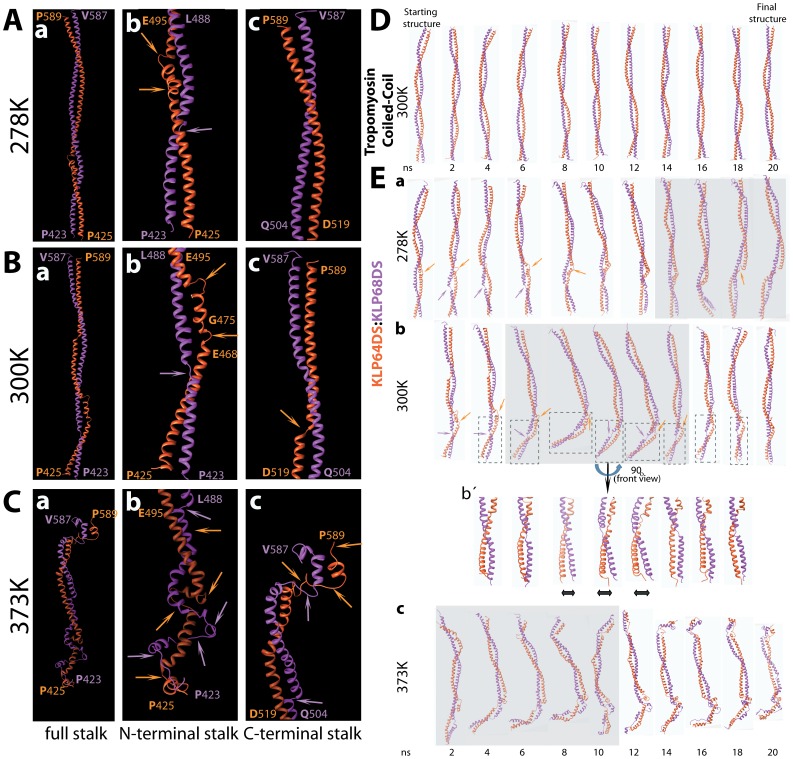
Interactions between the C-terminal regions of the KLP64D and KLP68D-Stalk polypeptides play a critical role in maintaining the heterodimer. A–C) Snapshots of KLP64D/68D-S heterodimer after 20 ns of MD simulations at 278K (A), 300K (B) and 373K (C), respectively. Ribbon structures of the full length stalk heterodimer (a), the N-terminal segments (P425–L506 of KLP64D and P423–I501 of KLP68D) (b), and the C-terminal segments (R517–P589 of KLP64D and T511–V587 of KLP68D) (c), respectively, are depicted in separate panels. Arrows indicate the unstructured regions in between the α-helices in KLP64D (orange arrow) and the KLP68D (magenta arrow) chains. D) Snapshots of the evolution of the tropomyosin coiled-coil structure during the MD simulations at 300 K. E) Snapshots of the evolution of the KLP64D/68D-Scoiled coil model at 278 K (a), 300 K (b and b´), and 373 K (c), respectively. The boxed region highlights dynamic fold-back of the N-terminal KLP64D/68D-Stalk at 300 K. An enlarged view of the N-terminal portion is shown in (b´) after a 90° rotation of the viewing angle. Arrows indicate the unstructured fragments present in between the α-helices in KLP64DS (orange arrow) and the KLP68DS (magenta arrow) chains. The shaded background box indicates simulation times when the stalk domain is in incomplete fold back configuration.

The radius of gyration (Rg) for both the proteins did not alter significantly at 300 K (Table 2), implying that both the CC and KLP64D/68D-S would remain extended (does not fold back and/or fully unfold) in water at ambient temperature. These changes were due to alterations in the α-helicity of individual backbones, occurring without affecting the total stabilization energy and the coiled-coil stability (Figure 4B and D). Similar variations in backbone RMSD with a comparatively lesser frequency were observed in KLP64D/68D-S simulations (Figure 3E-b and F-b, red arrows), suggesting the presence of coiled-coil structures in the heterodimer. Interestingly, the RMSD values of KLP64D/68D-S progressively increased up to 2 nm during the first 10 ns, and subsequently restored back to 0.6 nm by 14 ns and continue to fluctuate within a smaller range during the rest of the simulation (Figure 3E and F, red open arrows; and Figure 4E). These fluctuations were caused due to a reversible bending of the N-terminal parts of the constituent chains in the heterodimer. In comparison, the RMSD of CC oscillated between 0.4 and 1 nm during the entire simulation (Figure 3E and F, black arrows). Thus, the MD simulations suggested that KLP64D/68D-S would consist of partly coiled-coil structures with some unique, dynamic features. In spite of this dynamic nature of the stalk model, several hydrophobic, as well as charged interactions, distributed along the entire stalk between the KLP64DS and KLP68DS polypeptides, were retained during the simulations (Table 2, Table S3). This could contribute towards maintaining a stable association between the stalks even under stress as observed earlier. For instance, we found that, in spite of the fragile nature of the association, the His-KLP64D/68D-S could be purified by gel filtration, and the CD analysis showed that the heterodimer would retain some secondary structure at 90°C (Figure 2).

### KLP64DS Chain is Relatively more Flexible than KLP68DS Chain in the Heterodimer Model

A comparison of the root-mean-square fluctuations (RMSF) of Cα atoms of KLP64DS and KLP68DS residues at 300 K, with their corresponding chains in the CC model, suggested that some of the loops in both the chains, fluctuate significantly at 300 K (Figure 3E and F). Certain stretches of the KLP64DS chain in the heterodimer became flexible (residues 425A–445N, 483Q–513K, and 572G–589P) as compared to the CC chain A. In comparison, only two short stretches at the N (423P–444E) and C-termini (567E–584P) of the KLP68DS chain also became flexible. These stretches lie within the predicted coils 1 and 2, respectively (Figure 3E and F). Moreover, time evolutions of the secondary structure elements of the stalk domains suggested that more number of residues were present in the unfolded structures in KLP64DS as compared to the KLP68DS (Figure 4, Figure S4 and S5). The regions in KLP68DS, which complement the unfolded regions of KLP64DS, are structurally stable α–helices (Figure 3 and 4). Thus, consistent with the biochemical observation, the modelling also suggested that KLP64DS is inherently unstable, and it is stabilized in the heteroduplex with KLP68DS through distributed hydrophobic interactions and salt-bridges along the entire stalk. It also explained how KLP64DS could selectively form a heterodimer with KLP68DS. The flexibility of KLP64DS polypeptide chain may also have functional significance. For instance, the hinge region (468E–475G) formed at the N-terminus of the KLP64DS would provide flexibility to the twist angle between KLP64D and KLP68D stalk, specifically at the N-terminal region adjacent to neck sequences (Figure 4E). Earlier reports suggested that enhanced protein flexibility could improve their adaptability to temperature fluctuations [25]. For instance, enhanced flexibility of a few residues in the loop regions in psychrophilic trypsin at room temperature contributes to the functional adaptability of the enzyme [26]. Thus, more flexibility of some of the regions in KLP64DS (at 300 K) could be a positive factor contributing to greater conformational-adaptation during kinesin-II functions.

### KLP64D/68D-S Model after Solvent Relaxation - only the C-terminal parts Remain Engaged in a Coiled-coil, the N-termini of Individual Polypeptide Chains Unfold

**Figure 5:**
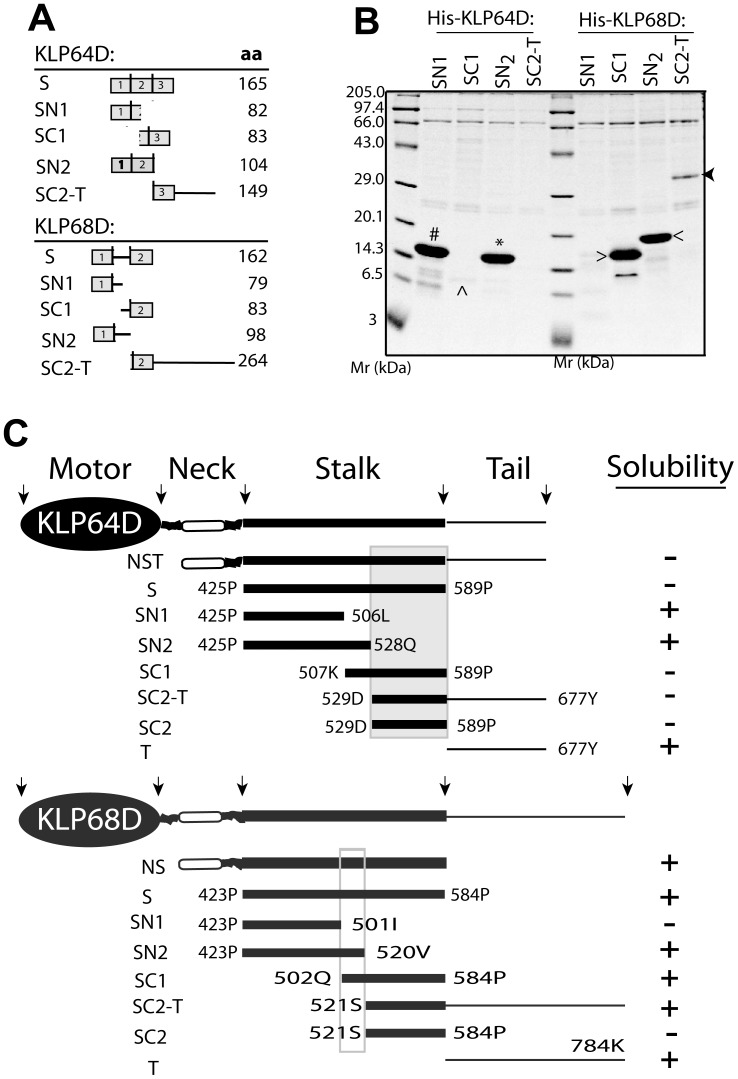
Expression and purification of the recombinant kinesin-II stalk fragments indicates complementary segmental stabilities in the stalk domains. A) Schematic illustrates the extents and corresponding lengths in amino acids (indicated under ‘aa’ on the right margin) of different stalk fragments cloned (Table S1). B) Equal amounts of extracts from *E. coli* expressing different recombinant stalk fragments were purified through Ni-NTA chromatography. The eluates were separated by SDS-PAGE and Coomassie stained to reveal the contents. Prominent bands of sizes expected for His-KLP64D-SN1 (#) and –SN2 (*), His-KLP68D-SC1 (>) and –SN2 (<), a faint His-KLP64D-SC1 (^∧^) band and a relatively weak His-KLP68D-SC2-T band (arrowhead) are marked on the figure. C) Schematic summary of the stalk fragment-stability. Grey box highlight the region imparting instability to KLP64DS and the open box indicates the region imparting stability to the KLP68DS. Abbreviations: NST – neck stalk tail, NS – neck stalk, S – stalk, SN1– N-terminal half of stalk, SN2– N-terminal 2/3^rd^ stalk, SC1– C-terminal half of stalk, SC2-T – C-terminal 1/3^rd^ stalk and tail, SC2– C-terminal 1/3^rd^ stalk, T- tail.

Both CD analysis and MD simulation of KLP64D/68D-S indicated that only a part of the heteroduplex would form a coiled coil. To test this conjecture, we calculated the solvent relaxed structures of different segments of KLP64D/68D-S. It showed that the α-helix contents in the N-terminal regions (SN1 and SN2) of both KLP64DS and KLP68DS reduced significantly, as compared to that of the C-terminal regions (SC1 and SC2) (sequence details of the fragments are provided in Figure 5C), at 278 and 300K, respectively. Few small stretches in the N-terminal regions of the both KLP64DS and KLP68DS unfold at 300 K (Table 3 and 4). However, the regions in KLP68DS, complementary to the unfolded stretches of KLP64DS, remained structurally stable, and *vice versa* (Figure 4). Interestingly, the secondary structures of the C-terminal regions remained mostly unaffected even at 373 K (Table 3 and 4; Figure 4). This clearly suggested that the secondary structures and the interchain interactions in the C-terminal region of KLP64D/68D-S heterodimer would be relatively more stable than the N-terminus. Moreover, a few interactions, such as the salt-bridges between KLP64D-R517 and KLP68D-E506, KLP64D-E562 and KLP68D-R559 remained unbroken even at 373K (Figure S6, Table S4), implying that coulomb interactions between the C-terminal halves keep the chains together. Time evolution analysis at 300 K further indicated that along with the unfolding of unstable helices, some of the loops also folded into helical forms (Figures S4 and S5).

**Table 4:** Average % α-helicity in different segments of the stalk domains of KLP64D and KLP68D after 20 ns MD simulations for different temperatures (averaged over last 100 ps).

	Temp (K)	SN1		SC1		SN2		SC2	
		64D	68D	64D	68D	64D	68D	64D	68D
% α-helix	278	89	86	98.8	97.6	91.4	88.8	96.7	96.9
	300	76.8	82.3	98.8	95.2	81.7	85.7	96.7	93.8
	373	63	64.5	72	75.9	71.2	71.4	62.3	68.5

Abbreviations: SN1– N-terminal half of stalk, SN2– N-terminal 2/3^rd^ stalk, SC1– C-terminal half of stalk, SC2– C-terminal 1/3^rd^ stalk (*please see* *Figure 5* *for stalk fragment details*).

At 373K, however, only a part of the stalk was unfolded (Figure S4 and S5). Once again, this matched the thermal unfolding data, which also suggested a residual structure even at 363 K. In addition, meticulous analysis of KLP64D/68D-S structures at different time points of MD simulations suggest that the 468E–475G segment of KLP64D would act as a hinge and provides flexibility to the N-terminal part of KLP64DS (Figure 4). A similar break was observed in the corresponding region of KLP68DS, as well. This hinge plays a critical role in providing dynamicity to the N-terminus of KLP64D/68D, which in turn may play a critical role in the corresponding motor walk. Thus, KLP64D/68D-S appeared to have a flexible N–terminal region, and the C-termini formed a relatively stable coiled coil. These features are distinct and not reported for any coiled coil structure so far.

### C-terminal Region of KLP64DS Imparts Instability and Middle Region of KLP68DS Provides Stability to the Individual Stalks

MD simulation showed that the KLP64D/68D-S model adopted a distinct and different form after the simulations compared to that of the tropomyosin coiled coil (PDB:1C1G), although the later was used as a starting template. Subsequent data analysis provided some new insights such as the flexibility of the N-terminal domain and complementary segmental instability. These possibilities were further tested by copurification studies and CD analysis. For this, we cloned multiple N- and C-terminal stalk fragments and expressed them in *E. coli* (Figure 5A). This showed that N-terminal KLP64D stalk fragments (-SN1 and -SN2), KLP68D-SN2 and KLP68D-SC1 fragments were soluble (Figure 5B); KLP68D-SC2-T was weakly soluble; and, KLP68D-SN1 was insoluble (Figure 5B). Several attempts to purify C-terminal fragments (SC2) of both KLP64D and KLP68D stalks were unsuccessful. A majority of the affinity purified soluble fragments eluted in volumes corresponding to the expected sizes of the monomer by gel filtration chromatography (Figure S7), indicating that some of the predicted coiled-coil segments such as the N-terminal coil-1 of KLP64DS could independently fold into a soluble form, whereas both the coil-1 and -2 of KLP68DS misfolded in the absence of the middle portion. Thus, the KLP68DS middle region (Q502–V520), with no predicted coiled-coil propensity, appeared to stabilize the polypeptides. In contrast, the C-terminal coil-3 (SC2: D529–P589) of KLP64DS was unstable and conferred the overall instability to the entire stalk. This complementary segmental stability (Figure 5C), therefore, could induce a cooperative folding of the two stalk fragments and act as a selection filter for heterodimerization.

### KLP64D and KLP68D Stalks Heterodimerize through the C-terminal 1/3^rd^ Segments, and the Association is Stabilized by the N-terminal Segments

**Figure 6:**
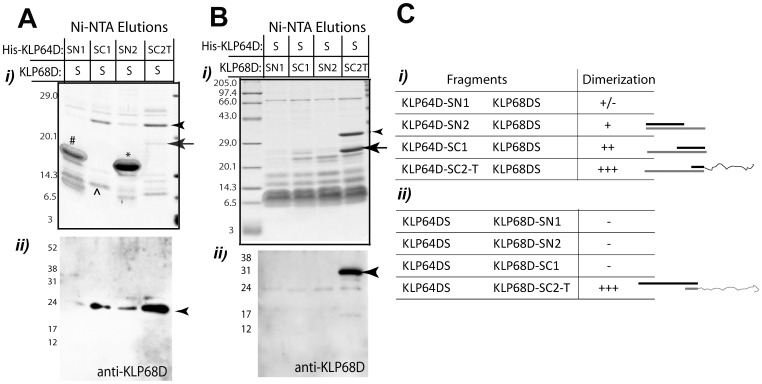
Combinatorial expression and pull-down analysis of full stalk and stalk fragments of kinesin-II motor subunits. A) 6xHis tagged KLP64D stalk fragments were coexpressed in *E. coli,* along with the KLP68DS, and purified by Ni-NTA chromatography, and separated by SDS-PAGE. *i*) Coomassie staining of SDS-PAGE gel of the Ni-NTA eluates. *ii*) Anti-KLP68D staining of the western blot of a similar gel indicates the presence of the KLP68DS bands (arrowhead) in different lanes. His-KLP64D-SN1 (#), -SC1 (^∧^) and –SN2 (*) bands; a relatively weak His-KLP64D-SC2-T band (arrow); and KLP68DS (arrowhead) are labeled. B) A similar experiment was performed with extracts of the *E. coli* coexpressing the His-KLP64DS and different KLP68D stalk fragments as indicated in the top panel. The arrow indicates His-KLP64DS band in (*i*) and the arrowheads indicates the KLP68D-SC2-T bands in both (*i*) and (*ii*). Since His-KLP64DS is only stable in a heterodimer with KLP68DS, the band was only visible in the last lane where it copurified KLP68D-SC2-T. C) The pull down results presented in panels A and B are summarized in tables (*i*) and (*ii*) respectively. Heterodimer formation is represented by ‘+’ and ‘–‘ denotes absence of heterodimer formation. Cartoon next to the table represents the stalk fragments that form heterodimer.

**Figure 7:**
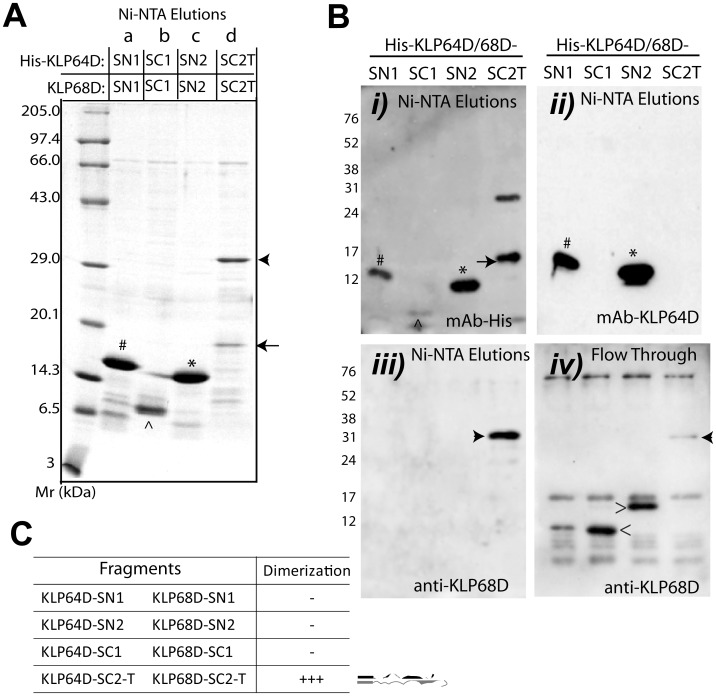
Combinatorial expressions and co-purification analysis of kinesin-II stalk fragments. A) Extracts of *E. coli* coexpressing, *a)* His-KLP64D-SN1 and KLP68D-SN1, *b*) His-KLP64D-SC1 and KLP68D-SC1, *c*) His-KLP64D-SN2 and KLP68D-SN2, and *d*) His-KLP64D-SC2-T along with KLP68D-SC2-T, were purified by Ni-NTA chromatography and separated by SDS-PAGE and stained with coomassie brilliant blue. B) Western blots of similar gels, as shown in (A), were stained with mAb-His (*i*), mAb-KLP64D (*ii*), and anti-KLP68D (*iii*), respectively. The unbound (flow through) fractions were blotted and stained with anti-KLP68D (*iv*). Bands corresponding to the expected sizes of His-KLP64D–SN1 (#),-SC1 (^∧^), -SN2 (*), and –SC2-T (arrow), as well as the KLP68D-SC1 (<), -SN2 (>) and SC2-T (arrowhead), are marked in the figures. In a separate study, mAb-KLP64D was found to stain the KLP64D-SN1 and –SN2 but not the C-terminal fragments, which is further confirmed by this data. C) Summary of the pull down results of the stalk fragments. Heterodimer formation is represented by ‘+’ and, ‘–‘ denotes absence of heterodimer formation. Cartoon next to the table represents the stalk fragments that form heterodimer.

The following, combinatorial coexpression and affinity copurification analyses of the stalk fragments revealed that two different C-terminal fragments of KLP64DS (-SC1 and -SC2-T), as well as the KLP64D-SN2, could pull down the full length KLP68DS (Figure 6A), whereas only the KLP68D-SC2-T copurified with the His-KLP64DS (Figure 6B). This association was independent of the His-tag position (Figure S8). A further paired coexpression of the equivalent KLP64DS and KLP68DS fragments showed that only the SC2-T fragments could form a stable association with each other (Figure 7A and B), and there was no affinity between the N-terminal fragments (Table 5). We also noticed that the N-terminal fragments of KLP68DS failed to associate with the full length KLP64DS, indicating that interaction between the C-terminal parts of KLP68DS and KLP64DS is essential to stabilize the complex. In addition, both the His-KLP64D-SC1 and His-KLP64DS could pull-down a full length KLP68DS, but they failed to pull-down KLP68D-SC1, indicating that the middle part of KLP68DS could potentially destabilize the association, which is somehow neutralized by the presence of the N-terminal half. Finally, we noted that the KLP64D/68D-SC2-T heteroduplex was stable (Table 5), suggesting that the presence of the tails might also stabilize the complex.

**Table 5:** Summary of the results of the combinatorial pull-down experiments.

Clone (MCS-1/MCS-2)	KLP64D	KLP68D	Heterodimer
His-KLP64D/68D-S	S	S	++++
HisKLP64D(SN1)/68D(S)	SN1	S	+/−
His-KL64D(SC1)/68D(S)	SC1	S	++
His-KLP64D(SN2)/68D(S)	SN2	S	+
His-KLP64D(SC2T)/68D(S)	SC2-T	S	+++
His-KLP64D(S)/68D(SN1)	S	SN1	–
His-KLP64D(S)/68D(SC1)	S	SC1	–
His-KLP64D(S)/68D(SN2)	S	SN2	–
His-KLP64D(S)/68D(SC2-T)	S	SC2-T	+++
His-KLP64D/68D-(SN1)	SN1	SN1	–
His-KLP64D/68D-(SC1)	SC1	SC1	–
His-KLP64D/68D-(SN2)	SN2	SN2	–
His-KLP64D/68D-(SC2-T)	SC2-T	SC2-T	+++

The full length stalk and stalk fragments pull down results presented in Figures 1, 6 and 7 are summarized here. The qualitative differences in the stability of the heterodimer are expressed in combinations of ‘+’ and/or ‘–’ signs. The numbers of signs are based on the efficiency of pull down observed between the respective fragments.

First, this analysis established that the stability of complementary fragments is not sufficient to induce an assembly in the absence of the C-terminal regions. Since the both KLP64D-SN1 and -SN2 failed to associate with the KLP68D-SN1 and –SN2, respectively (Table 5). Second, it indicated that interaction between the C-terminal one-thirds of KLP64DS and KLP68DS is the key to form a heterodimer with each other (Table 5). Third, it suggested that the N-terminal segments of the KLP68DS are required to stabilize the assembly. Moreover, presence of the tails may further stabilize the complex. Similar cooperative homodimer formation was observed in the NCD stalk where subdomain-1 was shown to depend on subdomains-2 and 3 for coiled-coil assembly [39]. This is remarkable since both the amino acid sequences and the heptad assignments in the kinesin-II stalks domains in *Drosophila*, *Xenopus* and *C. elegans* are highly divergent, but they display grossly similar heterodimerization properties [16], [19], [20].

### N-terminal Fragments of KLP64DS and KLP68DS are Flexible and do not form a Coiled Coil

**Figure 8:**
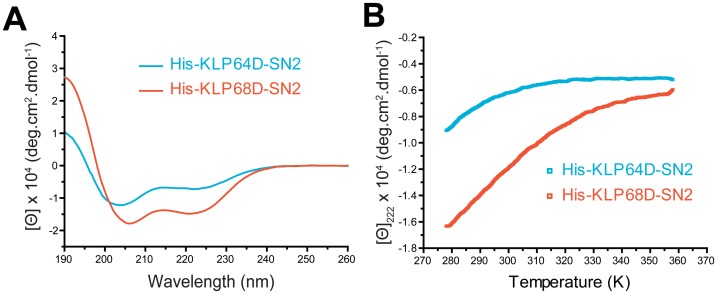
N-terminal 2/3^rd^ stalks (-SN2) of KLP64D and KLP68D contain low levels of alpha-helix and do not form coiled coils. A) CD spectra of His-KLP64D-SN2 (black) and His-KLP68D-SN2 (grey) obtained at 5°C.B) Thermal denaturation profiles of His-KLP64D-SN2 (black) and His-KLP68D-SN2 (grey) monitored at 222 nm.

One of the key predictions of the combined CD and MD simulation analyses was that the N-terminal segments of the KLP64D/68D-S would be mostly unfolded at ambient temperature. This is also partly supported by the combinatorial pull down analysis described here. Therefore, to test the hypothesis further, we studied the secondary structures of purified N-terminal stalk fragments. This revealed low alpha helix contents in the His-KLP64D-SN2 (10.6%) and His-KLP68D-SN2 (23.4%), at 5°C (278 K) (Figure 8A). In addition, the [Θ]_222_/[Θ]_208_ ratios are 0.71 and 0.85, respectively, indicating that these are unlikely to form coiled-coils on their own. The structures were also unstable and rapidly unfolded in 5–30°C (278–303 K) range (Figure 8B). Although these do not rule out the possibility of a coiled-coil association between the N-terminal fragments, induced in the presence of the C-terminal coils, it could suggest that the structural instability of the constituent chains would contribute to the structural dynamics of the KLP64D/68D-S heterodimer.

## Discussion

**Figure 9:**
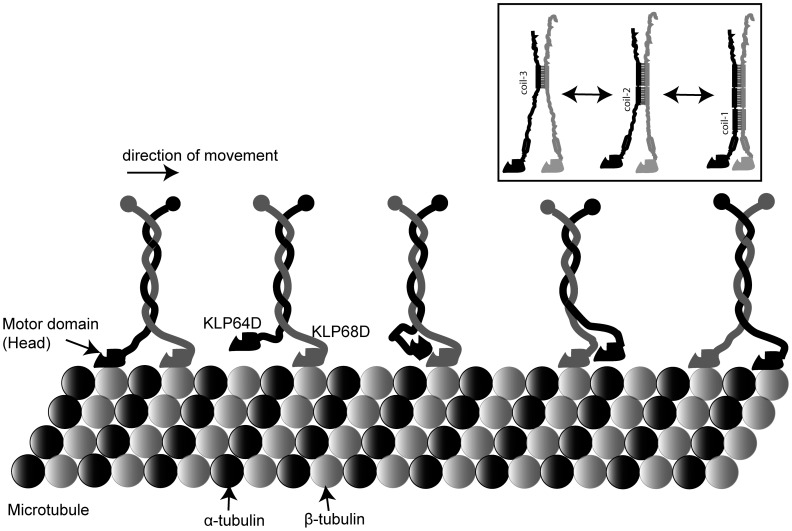
Kinesin-II stalk is a partially flexible coiled coil heterodimer. C-terminal ends of the kinesin-II stalks maintain a stable coiled coil link while the N-terminal segments reversibly alternate between coiled-coil and unfolded structures during the walk on a microtubule filament. Inset summarizes the reversible transitions of the N-terminal stalks as suggested by the results.

In summary, the comparative analysis of the KLP64D and KLP68D stalk fragments by a combination of biochemical, spectroscopic and bioinformatics tools showed that they form an unusual heteroduplex with part coiled coil structure at the C-terminal ends, whereas most part of the N-termini remained loosely attached (Figure 9). It is also evident that the association between the C-terminal parts is stabilized by distributed interactions along the length of the entire predicted stalk domain. Therefore, the heterodimeric kinesin-II stalk appeared to be distinctly unique as compared to the other kinesins. This study further provided MD simulation model for the stalks heterodimer and its predictions were further verified by CD experiments. They also opened the possibility to study the cofolding parameters of two independent polypeptide chains in solution and test the role of conserved charge interactions in the process. The relevance of these results on motor function is discussed below.

The MD simulations provided some intriguing insights. It showed that the N-terminal parts of the KLP64D/68D-S heterodimer could not retain the coiled coil structure, whereas the C-terminal and middle part of the stalk domain retain their coiled coil tertiary structure. Moreover, the 468E–475G region in the N-terminus of KLP64DS chain unfolded during the simulation stalk and behaved as a hinge in the stalk heterodimer, providing more flexibility to the N-terminal half of the stalk. In addition, while some stretches of α-helices retain their secondary structure; other parts unfolded during simulation. Therefore, the model for KLP64D/68D-S after 20 ns of simulation became markedly different from the starting model. This indicated that the simulation method could be used to gather coarse-grain, macroscopic views the of dynamic protein structures.

### Kinesin-II Stalks Fold Together through Complementary Stability

The coiled-coil assembly is often initiated from a consensus *trigger* sequence consisting of combination of charged and hydrophobic residues in certain heptad positions [21], [46], which could also help in registering the two participating polypeptides in a parallel order. The stalks of kinesin-II motor subunits do not contain a consensus *trigger* sequence, the C-terminal parts of the predicted kinesin-II stalk domains are essential for assembling a parallel coiled coil heteroduplex not only in *Drosophila* but also in *Xenopus* [16] and *C. elegans* [20], in spite of their poorly conserved amino acid sequence. The MD simulation and fragment copurification data presented above further indicate that interaction between charged and polar residues in ‘*a*’ and ‘*d*’ positions in the heptads could initiate and stabilize the KLP64D/68D-S assembly in a cooperative fashion. A similar process was observed with modified GCN-4 peptide in lipid environment [47], in the fos-jun heterodimer [48], and in the NCD stalk homodimer assembly [39]. Interestingly, the yeast homeodomain protein MATα2 was shown to contain three atypical amino acids at ‘*a*’ positions, which favoured heterodimerization with MATa1, and their replacement with isoleucine (typical amino acid found at ‘*a*’ positions) favoured homodimerization [49]. MD simulations also suggest that the KLP64DS is more flexible than KLP68DS, and the coiled coil association is only limited to the C-terminal parts. In case of the α/β- tropomyosin heterodimer, the instabilities of individual homodimers were shown to drive the heterodimerization [50]. Finally, a study on the neck coiled coil fragment of *Drosophila* kinesin-I showed that such atypical heptads add mechanical stability to a coiled coil by providing a greater refolding barrier [44]. Thus, monomeric instability combined with atypical distribution of charged residues could initiate selective heterodimerization between the kinesin-II stalks.

Forster resonance energy transfer (FRET) analysis of kinesin-II stalk heterodimer in *C. elegans* suggested that the N-terminal stalks are held together, but the CD analysis indicated only the C-terminal region (and not the N-terminal region) would be coiled coil [20]. This study enabled us to address the issue with the help of MD simulations and CD analysis of N-terminal stalks. The MD simulation suggested that even though the secondary structure and interactions between the N-terminal stalks are flexible, the ends remained in proximity to each other even after 20 ns. We confirmed that the association between the C-terminal stalks is more stable as compared to the N-termini. These conclusions are consistent with observed low helical content of the full length stalk. This study suggests that the charged and hydrophobic interactions between the stalk polypeptides are distributed along the entire chain, which would enable the kinesin-II motor subunits to remain associated with each other under stress.

### The Implications of Relative Instability of the KLP64D/68D-S Heteroduplex for Kinesin-II Motor Functions

Processive motor proteins function as dimers, which enable alternate hand-over-hand movement of a single molecule [51]. Even the UNC104 motor, thought to be monomeric earlier, was also shown to dimerize at higher concentration [52]. All dimeric motors contain a flexible neck-hinge linker between the motor and coiled coil stalk. This plays a vital role in the motor function [44]. The *Neurospora* kinesin-1 (Nckin-1) contains a soft coiled-coil neck. Its replacement with a comparatively stable coiled coil resulted in reduced speed and run-lengths at increasing loads [53]. Similarly, duplication of the KHC neck length increased the motor processivity and run-length [54]. Therefore, flexible association between the neck-hinge domains and the aminoacid length of the neck-hinge are critical for the motor processivity.

Kinesin-II motor subunits have relatively shorter neck and stalk domains than the kinesin-1 family members, and this could play a pivotal role in determining the motor function. Here, we showed that N-terminal portion of the heteroduplex is unstable, but it remains together through distributed charge interactions and coiled coil association between the C-terminal stalk fragments. In addition, single molecule atomic force spectroscopy of KLP64D/68D-S heterodimer failed to elicit a measurable adhesion force between the two chains at room temperature (Doodhi H, Agarwal V, Koti ASR and Ray K, *unpublished*). Therefore, we propose that the N-terminal segment of the kinesin-II stalk would undergo dynamic transition between the coiled coil and unstructured forms during the motor walk (Figure 9). This would effectively extend the length of the flexible neck-hinge domain and allow processive runs of the motor. Contrary to the longstanding belief, however, the structural integrity of the coiled-coil is shown to provide a rigid link between the kinesin heads, which is essential to coordinate the head movements for a longer period [55]. Therefore, weak association between kinesin-II stalks could also reduce the run-length/processivity. Indeed *in vitro* measurements show that recombinant kinesin-II has relatively shorter run lengths and lower average speeds *in vitro* as compared to the kinesin-I [12], [56]. How then such an unstable motor undertakes long range axonal transport? The run length of chick brain kinesin-II preparations containing the accessory subunit is comparatively longer [57], indicating that the KAP binding to the coiled-coil stalk domain [13] could stabilize the assembly. It would be now useful to test this hypothesis.

## Materials and Methods

### Secondary Structure and Coiled Coil Prediction

Secondary structure of KLP64D and KLP68D was predicted using PSIPRED v3.0 [31], [32]. Coiled coil prediction was carried out using MultiCoil program [33], with window size 28, the interaction distances for dimeric score: 3, 4, 5 and for trimeric score: 2, 3, 4.

### Cloning, Expression and Protein Purification

Recombinant KLP64D and KLP68D stalk fragments were PCR amplified from the cDNA with appropriate sets of primers. They were cloned into either the multiple cloning site-1 (MCS1) of pETDuet™-1 vector (Novagen Inc. USA) in frame with the 6xHistidine (His) tag at the N-terminus, or into the MCS2 of the same vector with a stop codon at the end of desired ORF (Table S1). The fragments cloned into the MCS2 cassette had no tag. They could be copurified by affinity chromatography along with the His-tagged polypeptide (expressed from the MCS1 cassette), only if bound to each other. pETDuet-1 plasmids harboring two different fragments in MCS1 and MCS2 were transformed into *E. coli* BL21 (DE3) and purified using affinity chromatography and gel filtration as described in the following section.

The recombinant proteins were purified using a protocol described earlier [13]. In brief, the cloned plasmid DNA constructs were transformed into *E. coli* BL21(DE3) cells. *E. coli* suspension cultures carrying respective DNA constructs were grown at 37°C till OD600 reached 0.6 to 0.8 and then induced with 0.5 mM IPTG for 4 hours at 28°C. The cells were harvested and stored at –80°C. For purification of protein, the cells were resuspended in lysis buffer [20 mM Tris-Cl (pH 7.5), 5 mM MgCl_2_, 300 mM NaCl, 1mM of DTT, 20 mM Imidazole, 1% Triton X-100, 0.5 mg/mL lysozyme and protease inhibitor cocktail (Roche Gmbh)] 10 mL of lysis buffer per gram of cell pellet was used. The cells were sonicated briefly on ice and then centrifuged at 4°C for 45 min at 70000 *g*. Ni-NTA agarose (Qiagen Inc.) was used to purify the recombinant proteins from the supernatant. The Ni-NTA agarose beads were washed with wash buffer [20 mM Tris-Cl (pH 7.5), 5 mM MgCl_2_, 150 mM NaCl, 1 mM of DTT, 50 mM Imidazole and eluted with elution buffer [20 mM Tris-Cl (pH 7.5), 5 mM MgCl_2_, 250 mM NaCl, 1 mM of DTT, 300 mM Imidazole]. The purified proteins were assessed by SDS-PAGE, and, the concentration was estimated by Bradford method as well as by spectrophotometric absorption at 280 nm.

### Gel Filtration Analysis

Affinity purified proteins were analyzed by gel filtration by using HiLoad 16/60 Superdex™-75pg (GE Healthcare Ltd., USA) in TMN buffer [20 mM Tris-Cl, pH7.5, 5 mM MgCl_2_, 500 mM NaCl] containing 2.5 mM DTT (TMN-D) at 1 ml/min flow rate, and 1.5 ml fractions of the eluate were collected by using Frac™-920 (GE Healthcare Ltd., USA). The volume fractions were analyzed by SDS-PAGE, and the suitable ones were pooled and concentrated using Centricon® spin concentrators (Millipore (India) Ltd., India). For Circular Dichroism (CD) study, the buffer of the concentrated fractions was further changed to 10 mM sodium phosphate (pH 7.5) containing 0.2 mM DTT by using PD-10 desalting column (GE Healthcare Ltd., USA) as per standard protocols.

### Circular Dichroism Spectroscopy

Far-ultraviolet (Far-UV) Circular Dichroism (CD) spectra were collected by using JASCO™-810 spectropolarimeter equipped with a Peltier cell temperature controller (±0.2°C). The spectra was recorded from 190 nm to 260 nm at 0.1 nm intervals at a 20 nm/min scanning speed. The measurements were averaged for 3 scans. The raw CD data obtained in millidegree were converted to mean residue molar ellipticity [Θ] (deg×cm^2^×dmol**^−^** ^1^) using the following equation:
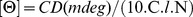
(1)Where *C* is the molar concentration, *l* is the path length in centimetres, and *N* is the number of amino acid residues present in the protein.

The secondary structure of the protein was estimated form the CD spectra in the far-UV region (190–260 nm) using Yang’s method [41]. The secondary structure was also estimated using Reed method [40] and a method used by De Marco et al., 2003 [19] for *Xenopus* kinesin-II stalks. The values obtained by Yang’s method was used in the manuscript, since the values estimated by Yang’s method and the method used by De Marco et al., 2003 matched. The secondary structure composition of proteins estimated by all the three methods could be found in Table S2.

### Thermal Unfolding

The temperature dependence of the CD spectra was monitored at 222 nm, with the sample placed in a cuvette of 0.1 cm path length. Temperature was raised from 5 to 90°C with a data pitch of 0.1°C along with simultaneous recording of the spectra at 2°C intervals from 5 to 25°C (278 to 298 K) and at 5°C intervals from 25 to ∼90°C (298 to ∼363 K). The protein unfolding results were analysed using a simplified apparent two state-equilibrium model between the folded (*N*) and unfolded (*U*) states:
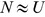
(2)


The fractions of the unfolded protein at different temperatures (T) were calculated using the following equation:
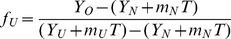
(3)


*Y_0_* is the observed mean residue molar ellipticity of the protein at 222 nm, at a given temperature (*T*); *Y_N_* and *Y_U_* represent the intercepts; and; *m_N_* and *m_U_* are the slopes of native and unfolded baselines.

The apparent equilibrium constant of unfolding 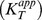 and the corresponding free energy change 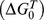
at temperature *T* was calculated using Equations (4) and (5):
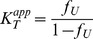
(4)

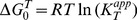
(5)


*R* is the gas constant; 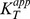 is the apparent equilibrium constant for the unfolding, and T is the absolute temperature.

The stability curves were obtained by non-linear least square analysis of 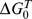 estimates by fitting it to the Gibbs-Helmholtz equation (Eq. 6). 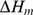 is the enthalpy change at the midpoint of transition, i.e., when *T = T_m_*. Therefore, at *T_m_*, 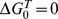, according to the following equation.
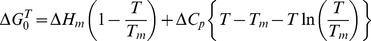
(6)


### Western Blots and Immunostaining

The purified recombinant proteins or pull-down products were separated by SDS-PAGE and transferred to a PVDF membrane (Hybond™-P, Amersham Biosciences Plc. UK) according to the suppliers protocol, and stained with antibodies as described before [13].

### Distant Homology Modeling of the Stalk Domains of Kinesin-II Motor Subunits and Molecular Dynamics Simulation

The secondary structure and coiled-coil probability of the KLP64D (AAF50786) and KLP68D (AAA50008) stalks were predicted/calculated using MultiCoil [49] and reaffirmed through Phyre 0.2 (www.sbg.bio.ic.ac.uk/phyre). The heterodimeric model of KLP64D and KLP68D stalk domains were built simultaneously using A and B chains (18% and 19% identities, respectively) of *Sus scrofa* tropomyosin homodimer (PDB code: 1C1G) as templates in MODELLER v8.2 (www.salilab.org/modeller) [53]. The generated model was ‘Powell’ energy minimized for 3000 iterations with ‘Tripos’ force field in SYBYL v7.1 (www.tripos.com). The details are mentioned in one of the previous article [13]. All molecular dynamics (MD) simulations were performed using the GROMACS 4.5.5 [45] package and GROMOS96 with 53A6 force field implemented on a LINUX architecture [58], [59]. The starting configuration for the reference pure coiled-coil was obtained from pig tropomyosin (PDB: 1C1G) homodimer with 284 residues per chain [60]. We have selected coordinates of 1–165 residues of the chain A and B of 1C1G (abbreviated as CC), and the KLP64D/68D-S model [13] as the starting structures. Thus, both the starting structures contained 330 residues. They were immersed in a triclinic box of SPC water molecules [61]. To neutralize the charge of the system 17 and 24 Na^+^ ions were added to KLP64D/68D-S and CC systems, respectively (Table S3). For solvent relaxation, each system was subjected to 50000 energy minimization steps by steepest descents and equilibrated for 100 ps in each two phases at different temperatures by position restrained molecular dynamics simulation. The equilibrated systems were then subjected to molecular dynamics simulations for 20 ns (20000 ps) each at 278 K, 300 K and 373 K. Periodic boundary conditions combined with minimum image convention were used under isothermal conditions using modified Berendsen thermostat algorithm [62], with relaxation times of 0.1 ps and, isobaric conditions using Parrinello-Rahaman algorithm with relaxation time 2 ps. LINCS algorithm was used to constrain bond length with a time step of 10 fs for all calculations. Long range electrostatic interactions were calculated using Particle Mesh Ewald (PME) with cubic interpolation. van der Waals and Coulombic interactions were truncated at 1 nm. The nonbonded pair list was updated every 5000 steps and conformations were stored every 10 ps during simulations. The geometric quality, secondary structure analysis and interface region of KLP64D/68DS heterodimer were analyzed using WHAT-IF (*swift.cmbi.ru.nl/servers*) and COILCHECK (http://caps.ncbs.res.in/coilcheck) servers. Other analyses were performed using scripts included with the GROMACS [45] distribution. The visual analysis of protein structures was carried out using Chimera [63].

## Supporting Information

Figure S1 Secondary structure predictions of Drosophila kinesin-II stalks. The amino acid sequences of KLP64D (A) and KLP68D (B) was analyzed using PSIPRED [31], [32]. Only the stalk portion of KLP64D (A) and KLP68D (B) which is rich in alpha helical secondary structure is displayed. The helical residues are denoted by ‘H’ and the respective confidence of prediction is indicated by blue bars. The beginning and end of the stalk residues are denoted by a red arrow in each case.
(TIF)

Figure S2 The coiled coils of kinesin-II stalks. The coiled coil forming regions in KLP64D and KLP68D are predicted using MultiCoil program [33]. The prediction was carried out with window size of 28, interaction distances for dimer was set to 3, 4, and 5, and for trimersat 2, 3, and 4. The amino acid positions are shown on the X-axis and the coiled coil probability score on Y-axis. The middle region P425 to P589 of KLP64D and P423 to P584 of KLP68D were predicted to form coiled coil (shown by red doubled headed arrows). The neck region of KLP64D has very low coiled coil forming propensity, whereas, that of KLP68D has no coiled coil forming propensity (shown by black double headed arrow).
(TIF)

Figure S3 Measuring the coiled coil propensity of KLP64D/68D-S. Far-UV CD spectroscopic analysis of His-KLP68D/64D-S (P2 form), was performed in phosphate (benign) buffer [10 mM sodium phosphate (pH 7.5), 0.2 mM DTT] and the same buffer containing 50% Trifluoroethanol (TFE), respectively. The helical content of His-KLP68D/64D-S in benign buffer (solid black line) is 43.7% and that in 50% TFE is 45.6% (dotted black line). The secondary structure was estimated using Yang method [41]. The [Θ]_222_/[Θ]_208_ ratio in 50% TFE decreased to 0.95 from 1.03 in benign buffer.
(TIF)

Figure S4 Distribution of α–helices and turns along the KLP64D stalk unfold at different temperature. Time evolution of the secondary structural elements of the KLP64D stalk domainin KLP64D:68D-S at 278 K (a), 300 K (b) and 373 K (c), presented at every 2 ns for 20 ns duration. Key: H-α-helix, B- β-sheet, T- turn, L- loop, gaps- undefined.
(TIF)

Figure S5 Distribution of α–helices and turns along the KLP68D stalk unfold at different temperature. Time evolution of the secondary structural elements of the KLP68D stalk domainin KLP64D/68D-S at 278 K (a), 300 K (b) and 373 K (c), presented at every 2 ns for 20 ns duration. Key: H- α-helix, B- β-sheet, T- turn, L- loop, gaps- undefined.
(TIF)

Figure S6 Salt bridge oscillations during MD simulation. Time evolution of the lengths of a few selected inter-chain salt bridges along the length of the stalk heterodimer at 278 K (black), 300 K (red) and 373 K (green).
(TIF)

Figure S7 Gel filtration analysis of the affinity purified stable stalk fragments of the KLP64D and KLP68D. Fraction-wise absorption profiles of the eluates were shown at the left panel. The Y-axis indicates absorptions at 280 nm with a vertical bar denoting 10 mAU (milli absorption units) and the dotted lines denote the base line of absorption. The grey bar denotes the elution volume in which the individual fragments were eluted. The Coomassie stained SDS-PAGE of representative fractions under each peak as marked by the numerals are presented in the right panel. Lane E was loaded with the affinity purified sample used for gel filtration.
(TIF)

Figure S8 Combinatorial pull down of kinesin-II stalk fragments with tags swapped. 6xHis-tag (H) is placed on either KLP64DS fragments or on KLP68DS to study the effect of 6xHis-tag on their association with each other. Coomassie stained SDS-PAGE gels indicate the composition of the affinity co-purification of KLP68DS with His-KLP64D-SC1 (lane 1), KLP64D-SC1 with His-KLP68DS (lane 2), KLP68DS with His-KLP64D-SC2-T (lane 3), and KLP64D-SC2-T with His-KLP68DS (lane 4).
(TIF)

Table S1 Detail list of recombinant fragments cloned. The recombinant DNA fragments were cloned in the bacterial expression vector pETDuet-1 (Novagen Inc., USA). The cloned position MCS1/2 in pETDuet-1 is indicated within parenthesis. Fragments cloned in MCS1 expressed with N-terminal 6xHis tag,and they are denoted with a ‘His-’ prefix, while those in the MCS2 have no such tag. For co-expression, one of the fragments was cloned in the MCS1 and the other in MCS2. In the text, these co-expression clones are denoted as His-X/Y, where ‘X’ is the fragment that was expressed with 6xHis tag from MCS-1, whereas ‘Y’ was expressed from MCS2 without any tag. *GST-KLP64D-NST was cloned in pGEX4T1 vector and ^#^ His-KLP68D-NS was cloned in pQE30 vector.
(DOC)

Table S2 Secondary structure estimations of kinesin-II stalks from the CD spectra. The secondary structure of the recombinant stalk fragments was estimated form the respective CD spectra obtained in the far-UV region (190–260 nm) at 278 K (5°C) by using three different methods as listed in the table. *-Helical content is calculated from the observed [Θ]_222_ divided by the predicted molar ellipticity×100. The predicted molar ellipticity is calculated form [Θ]_222_ = −40000×(1–4.6/n) for chain length dependence of a helix [19] [De Marco et al., (2003) EMBO Rep 4∶717–722], Where n is the number of residues in the protein.
(DOC)

Table S3 List of Parameters used for MD simulations.
(DOC)

Table S4 List of Salt-Bridges in KLP64D/68D-S after 20 ns at 300 K.
(DOC)
