# Supplementary figures and images for: Biochemical and Molecular Dynamic Simulation Analysis of a Weak Coiled Coil Association between Kinesin-II Stalks
Source: PLoS One. 2012 Sep 28;7(9):e45981. doi: 10.1371/journal.pone.0045981 (PMC3461054; doi:10.1371/journal.pone.0045981)

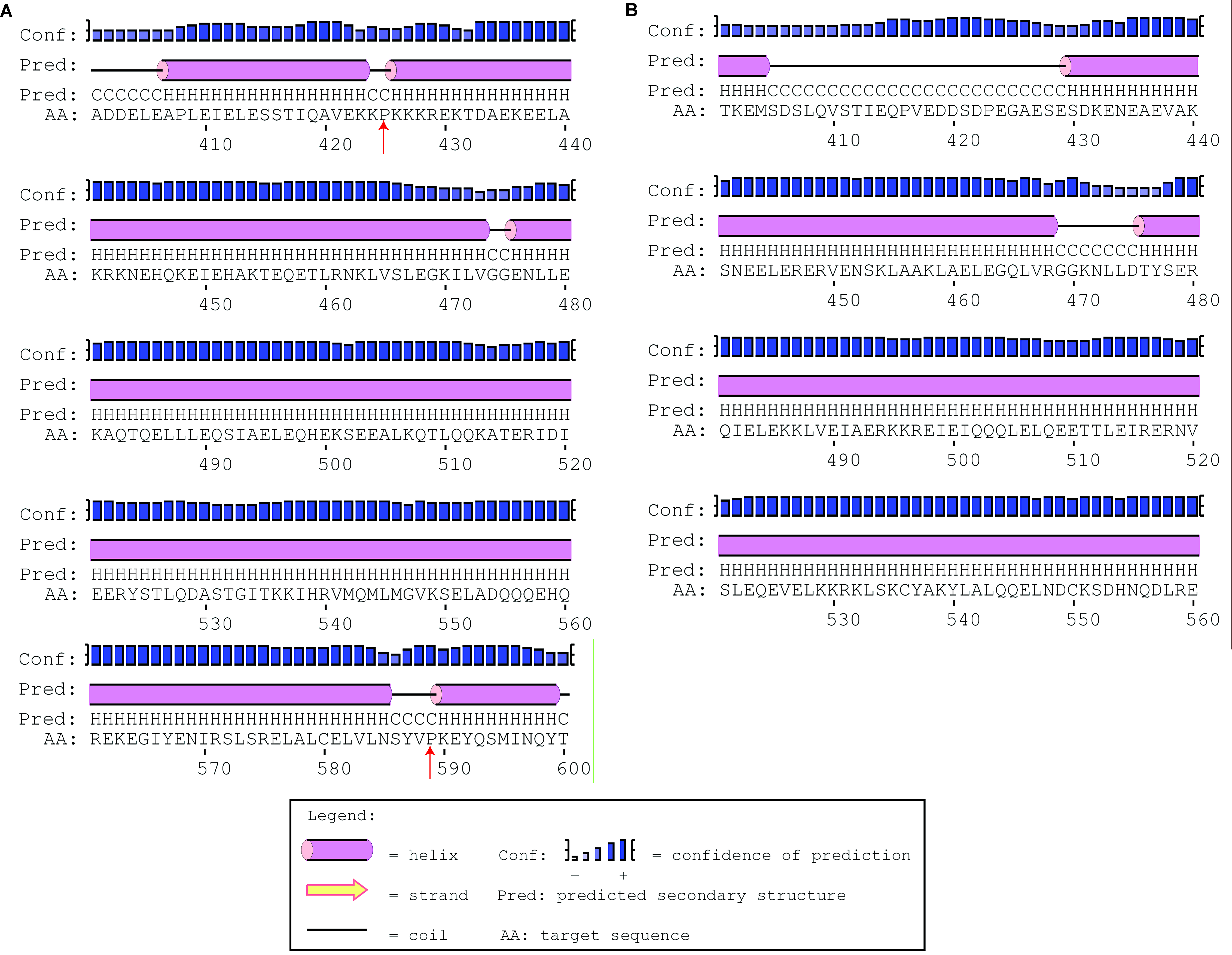

Supplement: Figure S1 — Secondary structure predictions of Drosophila kinesin-II stalks. The amino acid sequences of KLP64D (A) and KLP68D (B) was analyzed using PSIPRED [31], [32]. Only the stalk portion of KLP64D (A) and KLP68D (B) which is rich in alpha helical secondary structure is displayed. The helical residues are denoted by ‘H’ and the respective confidence of prediction is indicated by blue bars. The beginning and end of the stalk residues are denoted by a red arrow in each case. (TIF) [file pone.0045981.s001.tif]

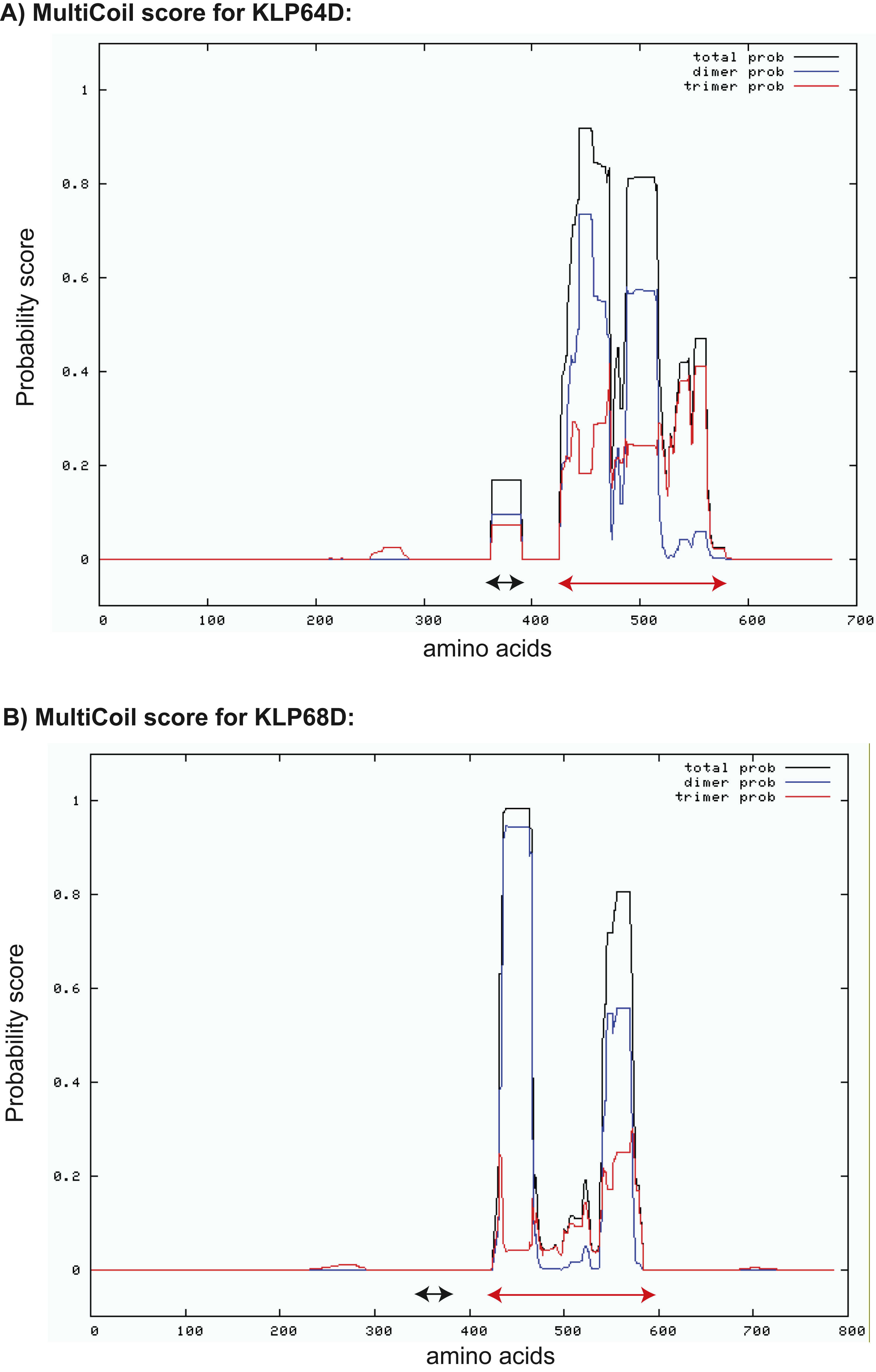

Supplement: Figure S2 — The coiled coils of kinesin-II stalks. The coiled coil forming regions in KLP64D and KLP68D are predicted using MultiCoil program [33]. The prediction was carried out with window size of 28, interaction distances for dimer was set to 3, 4, and 5, and for trimersat 2, 3, and 4. The amino acid positions are shown on the X-axis and the coiled coil probability score on Y-axis. The middle region P425 to P589 of KLP64D and P423 to P584 of KLP68D were predicted to form coiled coil (shown by red doubled headed arrows). The neck region of KLP64D has very low coiled coil forming propensity, whereas, that of KLP68D has no coiled coil forming propensity (shown by black double headed arrow). (TIF) [file pone.0045981.s002.tif]

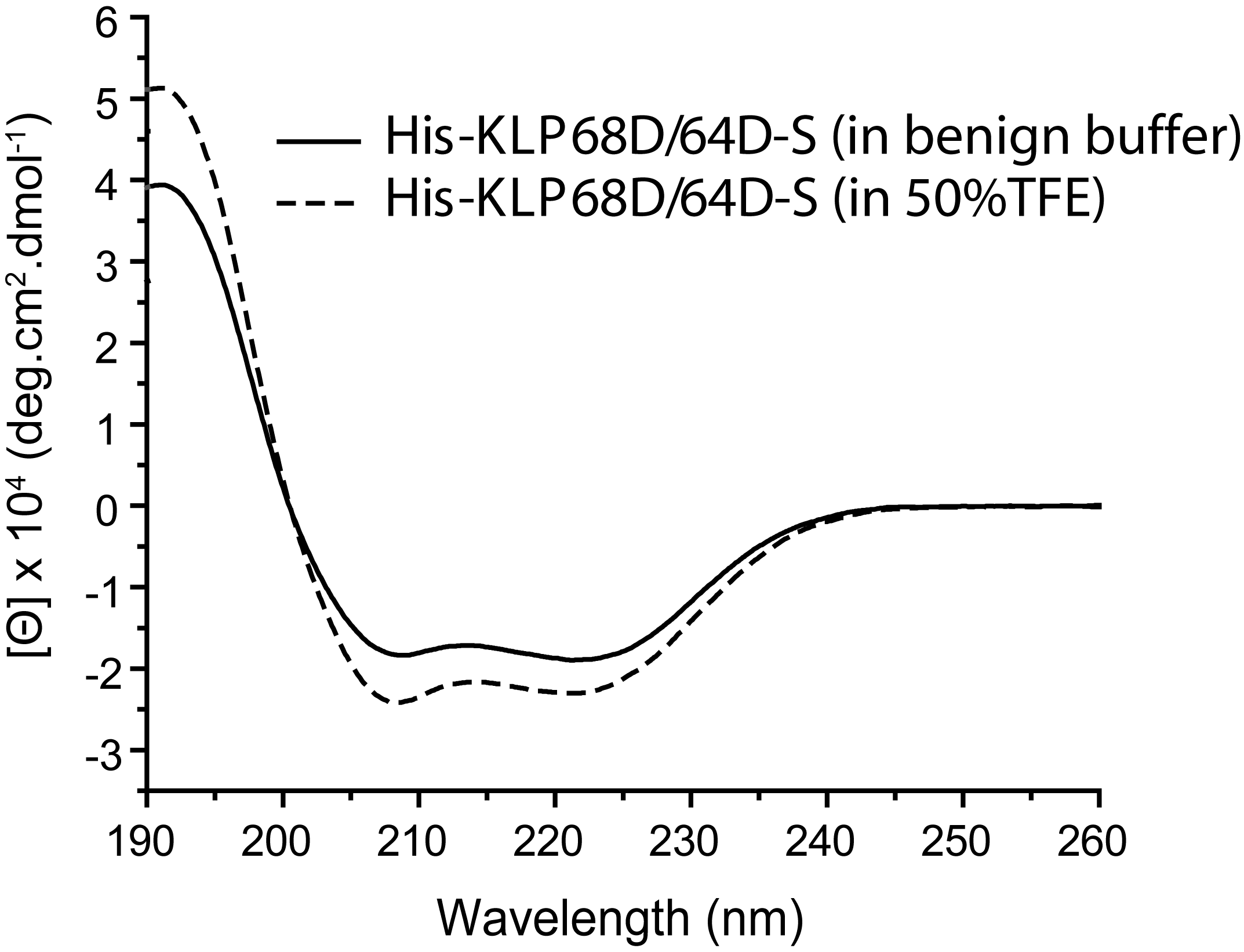

Supplement: Figure S3 — Measuring the coiled coil propensity of KLP64D/68D-S. Far-UV CD spectroscopic analysis of His-KLP68D/64D-S (P2 form), was performed in phosphate (benign) buffer [10 mM sodium phosphate (pH 7.5), 0.2 mM DTT] and the same buffer containing 50% Trifluoroethanol (TFE), respectively. The helical content of His-KLP68D/64D-S in benign buffer (solid black line) is 43.7% and that in 50% TFE is 45.6% (dotted black line). The secondary structure was estimated using Yang method [41]. The [Θ]222/[Θ]208 ratio in 50% TFE decreased to 0.95 from 1.03 in benign buffer. (TIF) [file pone.0045981.s003.tif]

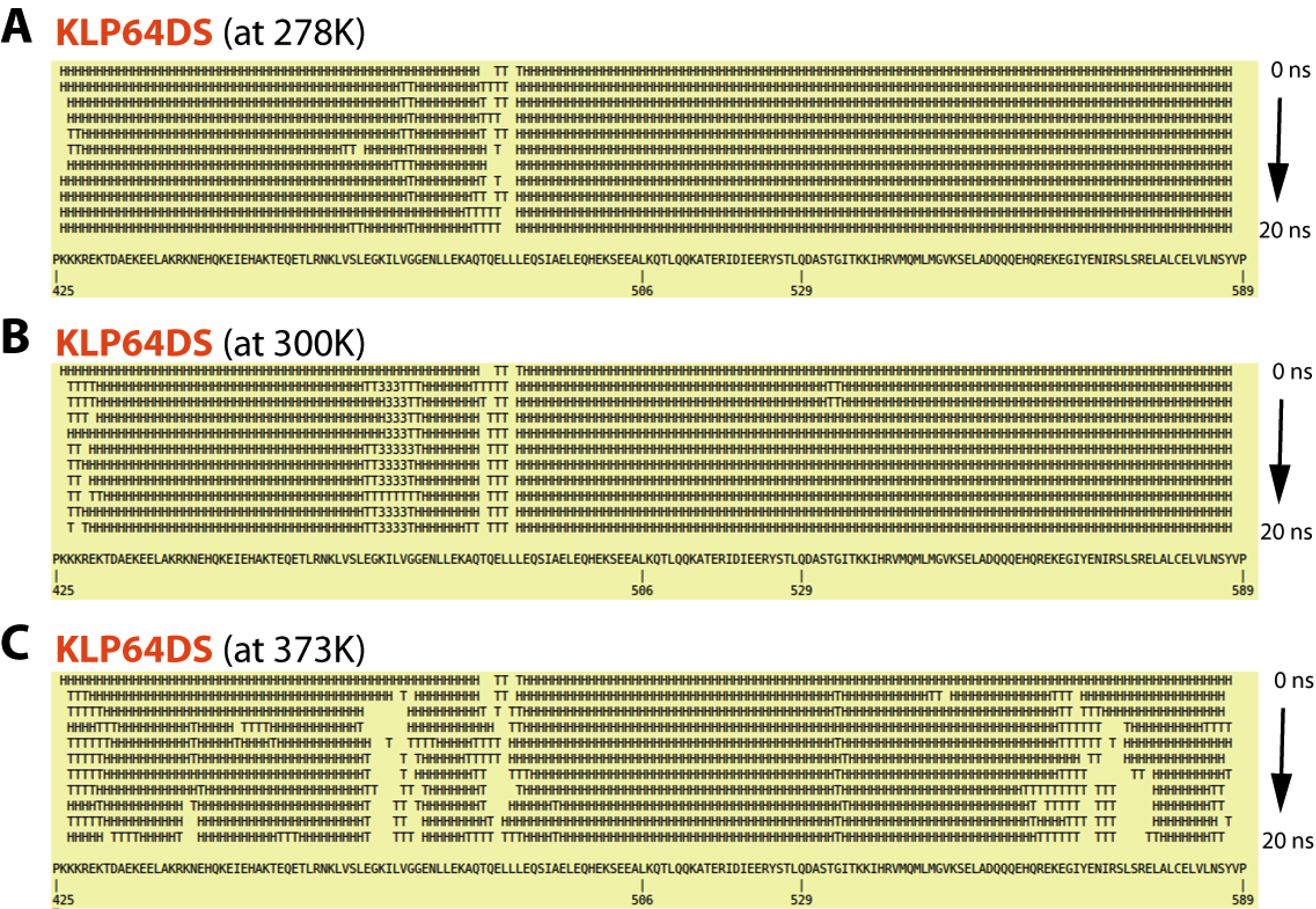

Supplement: Figure S4 — Distribution of α–helices and turns along the KLP64D stalk unfold at different temperature. Time evolution of the secondary structural elements of the KLP64D stalk domainin KLP64D:68D-S at 278 K (a), 300 K (b) and 373 K (c), presented at every 2 ns for 20 ns duration. Key: H-α-helix, B- β-sheet, T- turn, L- loop, gaps- undefined. (TIF) [file pone.0045981.s004.tif]

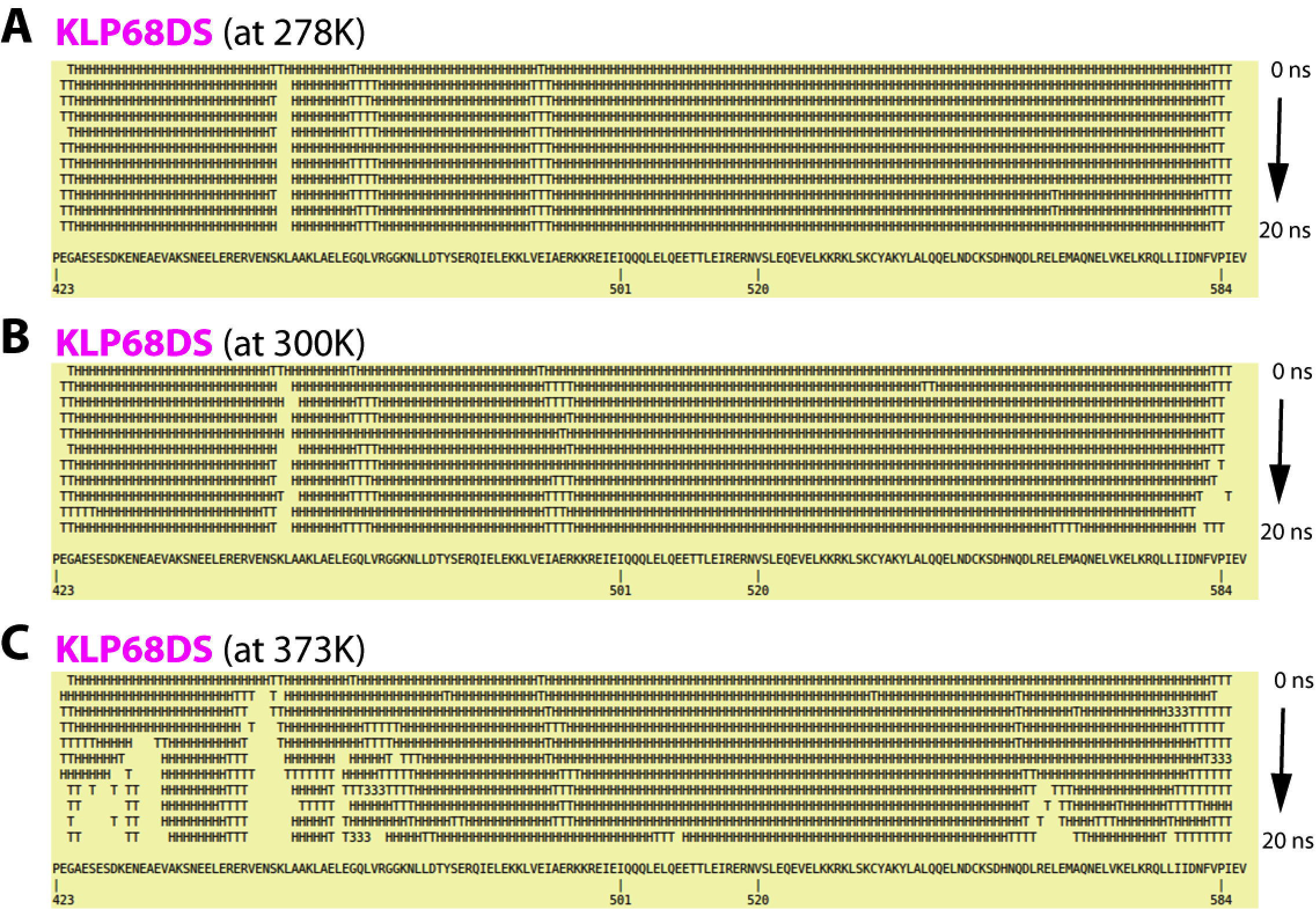

Supplement: Figure S5 — Distribution of α–helices and turns along the KLP68D stalk unfold at different temperature. Time evolution of the secondary structural elements of the KLP68D stalk domainin KLP64D/68D-S at 278 K (a), 300 K (b) and 373 K (c), presented at every 2 ns for 20 ns duration. Key: H- α-helix, B- β-sheet, T- turn, L- loop, gaps- undefined. (TIF) [file pone.0045981.s005.tif]

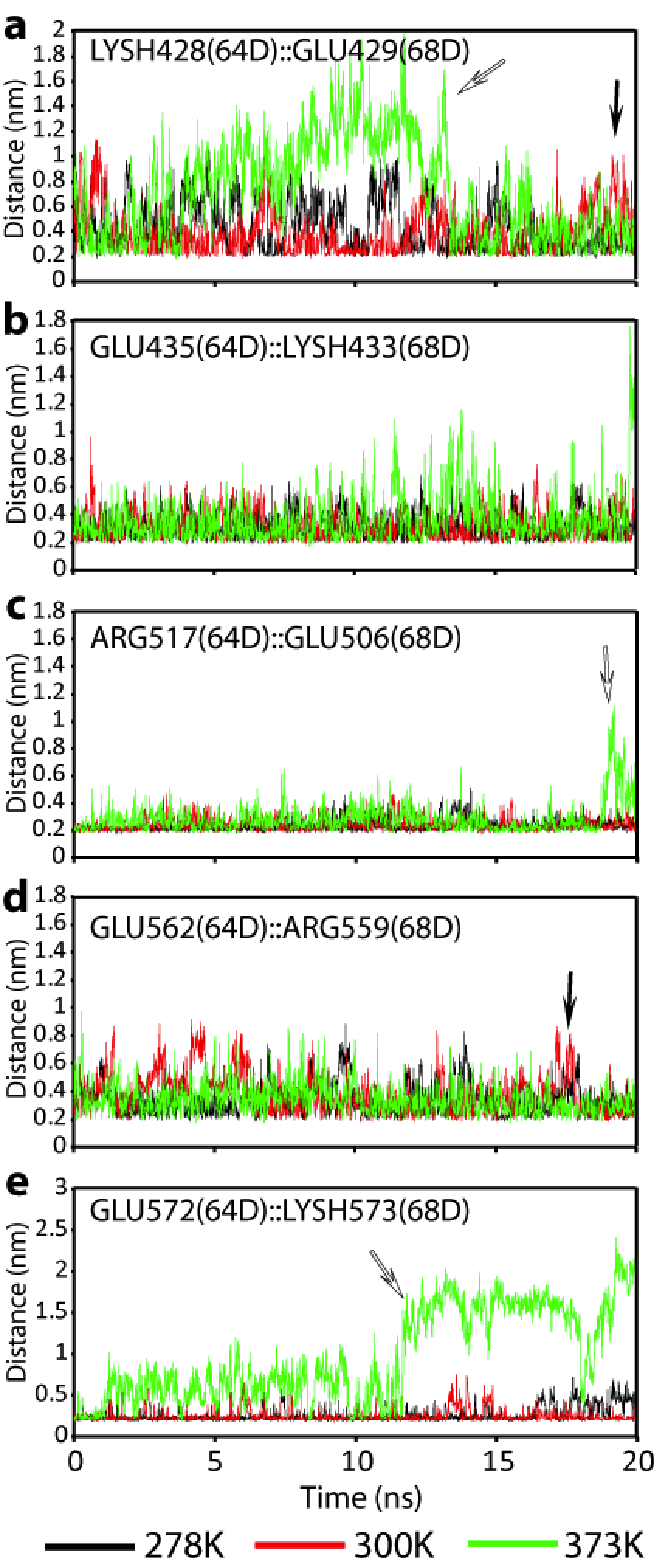

Supplement: Figure S6 — Salt bridge oscillations during MD simulation. Time evolution of the lengths of a few selected inter-chain salt bridges along the length of the stalk heterodimer at 278 K (black), 300 K (red) and 373 K (green). (TIF) [file pone.0045981.s006.tif]

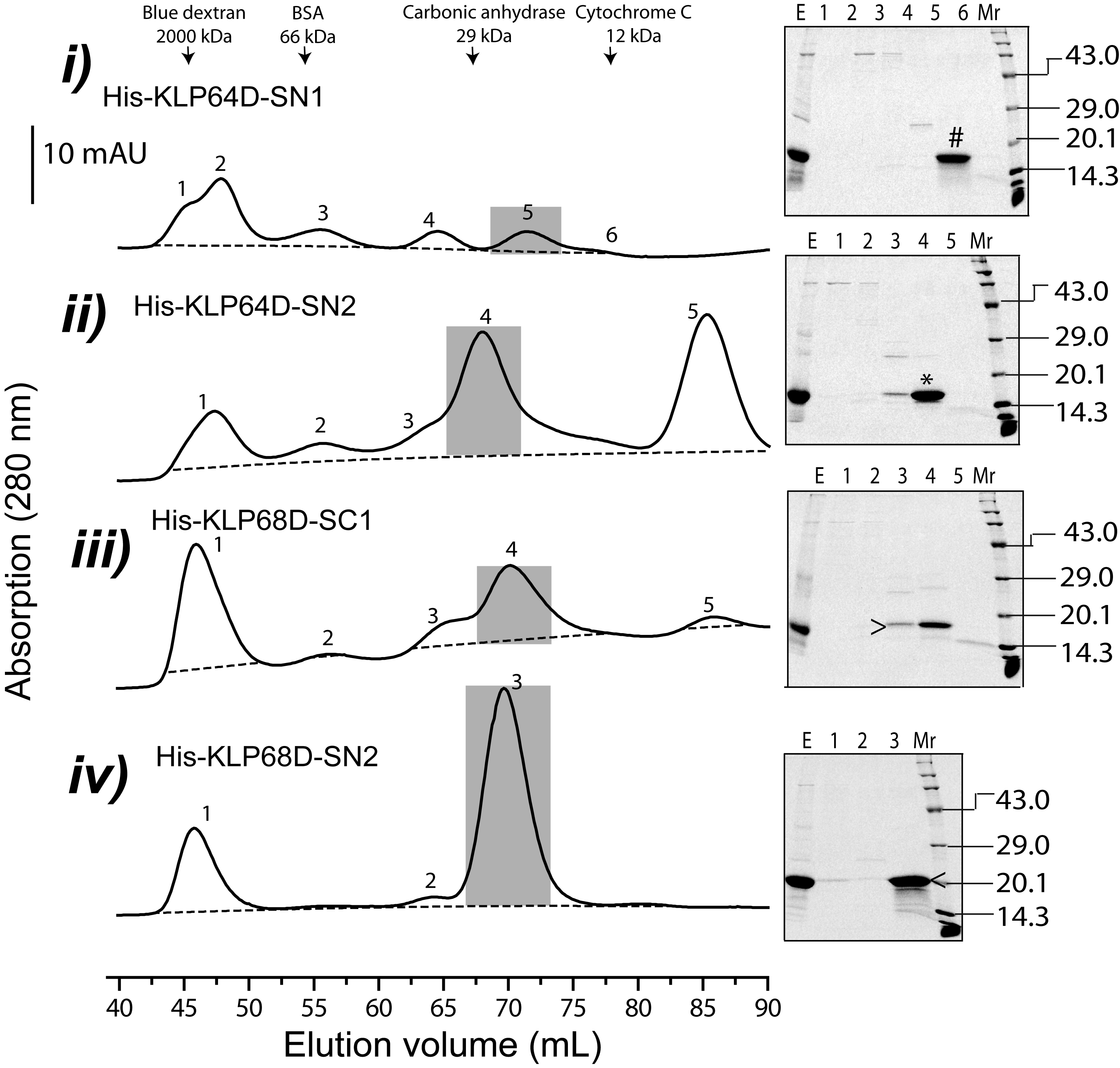

Supplement: Figure S7 — Gel filtration analysis of the affinity purified stable stalk fragments of the KLP64D and KLP68D. Fraction-wise absorption profiles of the eluates were shown at the left panel. The Y-axis indicates absorptions at 280 nm with a vertical bar denoting 10 mAU (milli absorption units) and the dotted lines denote the base line of absorption. The grey bar denotes the elution volume in which the individual fragments were eluted. The Coomassie stained SDS-PAGE of representative fractions under each peak as marked by the numerals are presented in the right panel. Lane E was loaded with the affinity purified sample used for gel filtration. (TIF) [file pone.0045981.s007.tif]

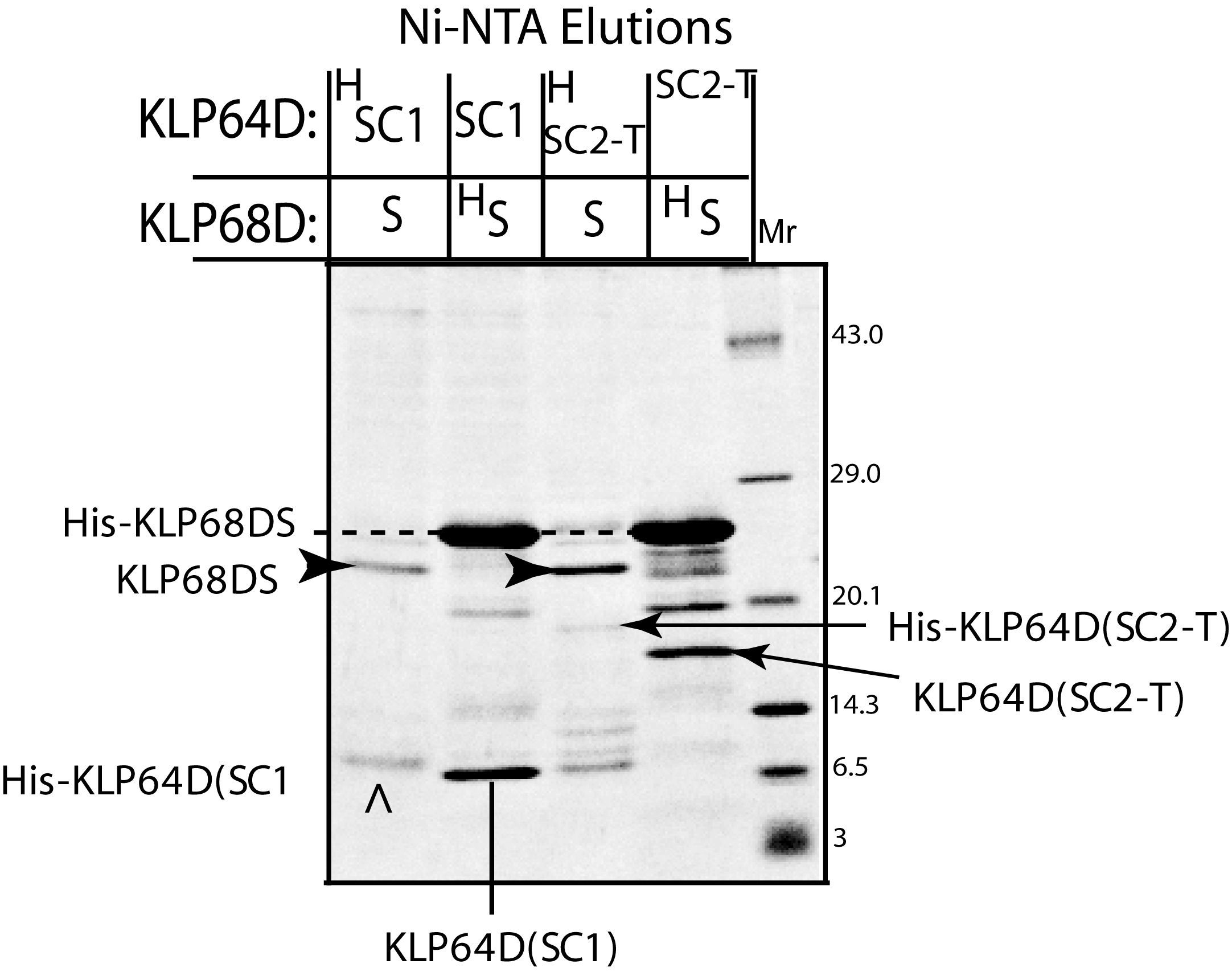

Supplement: Figure S8 — Combinatorial pull down of kinesin-II stalk fragments with tags swapped. 6xHis-tag (H) is placed on either KLP64DS fragments or on KLP68DS to study the effect of 6xHis-tag on their association with each other. Coomassie stained SDS-PAGE gels indicate the composition of the affinity co-purification of KLP68DS with His-KLP64D-SC1 (lane 1), KLP64D-SC1 with His-KLP68DS (lane 2), KLP68DS with His-KLP64D-SC2-T (lane 3), and KLP64D-SC2-T with His-KLP68DS (lane 4). (TIF) [file pone.0045981.s008.tif]
