# Supplementary material for: Biochemical and Molecular Dynamic Simulation Analysis of a Weak Coiled Coil Association between Kinesin-II Stalks
Source: PLoS One. 2012 Sep 28;7(9):e45981. doi: 10.1371/journal.pone.0045981 (PMC3461054; doi:10.1371/journal.pone.0045981)
Supplement: Table S1 — Detail list of recombinant fragments cloned. The recombinant DNA fragments were cloned in the bacterial expression vector pETDuet-1 (Novagen Inc., USA). The cloned position MCS1/2 in pETDuet-1 is indicated within parenthesis. Fragments cloned in MCS1 expressed with N-terminal 6xHis tag,and they are denoted with a ‘His-’ prefix, while those in the MCS2 have no such tag. For co-expression, one of the fragments was cloned in the MCS1 and the other in MCS2. In the text, these co-expression clones are denoted as His-X/Y, where ‘X’ is the fragment that was expressed with 6xHis tag from MCS-1, whereas ‘Y’ was expressed from MCS2 without any tag. *GST-KLP64D-NST was cloned in pGEX4T1 vector and # His-KLP68D-NS was cloned in pQE30 vector. (DOC) [file pone.0045981.s009.doc]

**Table S1. Detail list of recombinant fragments cloned:**

| **Cloned Insert and site in pETDuet^(R)^** | **Cloned DNA (bp)** | **Estimated length (aa)** | **Expressed size (kDa)** | **Positions** |
| --- | --- | --- | --- | --- |
| His-KLP64DS (MCS1) | 495 | 183 | 21.1 | P425-P589 |
| KLP64DS (MCS2) | 495 | 169 | 19.6 | P425-P589 |
| His-KLP68DS (MCS1) | 486 | 180 | 20.9 | P423-P584 |
| KLP68DS (MCS2) | 486 | 166 | 19.4 | P423-P584 |
| His-KLP64D-SN1 (MCS1) | 246 | 100 | 11.5 | P425-L506 |
| His-KLP64D-SC1 (MCS1) | 249 | 101 | 11.7 | K507-P589 |
| KLP64D-SC1 (MCS2) | 249 | 87 | 10.1 | K507-P589 |
| His-KLP64D-SN2(MCS1) | 312 | 117 | 13.7 | P425-Q528 |
| His-KLP64D-SC2-T(MCS1) | 450 | 162 | 18.7 | D529-Y677 |
| KLP64D-SC2-T (MCS2) | 450 | 153 | 17.6 | D529-Y677 |
| His-KLP68D-SN1(MCS1) | 237 | 97 | 11.0 | P423-I501 |
| KLP68D-SN1 (MCS2) | 237 | 83 | 9.5 | P423-I501 |
| His-KLP68D-SC1(MCS1) | 249 | 101 | 11.9 | Q502-P584 |
| KLP68D-SC1 (MCS2) | 249 | 87 | 10.4 | Q502-P584 |
| His-KLP68D-SN2MCS1) | 294 | 111 | 12.9 | P423-V520 |
| KLP68D-SN2 (MCS2) | 294 | 102 | 11.8 | P423-V520 |
| His-KLP68D-SC2-T(MCS1) | 792 | 277 | 30.9 | S521-K784 |
| KLP68D-SC2-T (MCS2) | 792 | 268 | 29.8 | S521-K784 |
| His-KLP64D-T(MCS1) | 267 | 101 | 11.7 | K590-Y677 |
| His-KLP68D-T(MCS1) | 600 | 213 | 23.2 | I585-K784 |
| *GST-KLP64D-NST | 1017 | 567 | 65.8 | 340I-674P |
| ^#^His-KLP68D-NS | 684 | 239 | 28.1 | Y362-I585 |
