# Supplementary material for: Biochemical and Molecular Dynamic Simulation Analysis of a Weak Coiled Coil Association between Kinesin-II Stalks
Source: PLoS One. 2012 Sep 28;7(9):e45981. doi: 10.1371/journal.pone.0045981 (PMC3461054; doi:10.1371/journal.pone.0045981)
Supplement: Table S2 — Secondary structure estimations of kinesin-II stalks from the CD spectra. The secondary structure of the recombinant stalk fragments was estimated form the respective CD spectra obtained in the far-UV region (190–260 nm) at 278 K (5°C) by using three different methods as listed in the table. *-Helical content is calculated from the observed [Θ]222 divided by the predicted molar ellipticity×100. The predicted molar ellipticity is calculated form [Θ]222 = −40000×(1–4.6/n) for chain length dependence of a helix [19] [De Marco et al., (2003) EMBO Rep 4∶717–722], Where n is the number of residues in the protein. (DOC) [file pone.0045981.s010.doc]

**Table S2.The secondary structure of the protein estimated form the CD spectra in the far-UV region (190-260 nm) at 278 K (5 ^o^C) using different methods:**

| Sample | helix | Beta-sheet | Turns | Random coil | NRMSD  (x100) |
| --- | --- | --- | --- | --- | --- |
| Reed Method [40] | | | | | |
| His-KLP64D/68D-S | 78.1% | 6.6% | 0.0% | 15.3% | 4.771 |
| His-KLP68D/64D-S | 95.7% | 0.0% | 0.0% | 4.3% | 4.071 |
| His-KLP64D-SN2 | 42.7% | 0.0% | 0.0% | 57.3% | 7.335 |
| His-KLP68D-SN2 | 60.0% | 0.0% | 1.7% | 38.2% | 5.543 |
| Yang Method [41] | | | | | |
| His-KLP64D/68D-S | 34.5% | 30.7% | 0.0% | 34.7% | 5.795 |
| His-KLP68D/64D-S | 41.4% | 23.6% | 0.0% | 35.0% | 5.768 |
| His-KLP64D-SN2 | 10.6% | 40.1% | 0.0% | 49.3% | 18.442 |
| His-KLP68D-SN2 | 23.4% | 33.8% | 0.0% | 42.9% | 11.913 |
| Ad hoc method used by De Marco et al., 2003 [19] (*) | | | | | |
| His-KLP64D/68D-S | 42.5% |  |  |  |  |
| His-KLP68D/64D-S | 42.9% |  |  |  |  |
| His-KLP64D-SN2 | 18.8% |  |  |  |  |
| His-KLP68D-SN2 | 38.83% |  |  |  |  |
