# Supplementary material for: Biochemical and Molecular Dynamic Simulation Analysis of a Weak Coiled Coil Association between Kinesin-II Stalks
Source: PLoS One. 2012 Sep 28;7(9):e45981. doi: 10.1371/journal.pone.0045981 (PMC3461054; doi:10.1371/journal.pone.0045981)
Supplement: Table S3 — List of Parameters used for MD simulations. (DOC) [file pone.0045981.s011.doc]

**Table S3. Parameters used for MD simulations:**

| Proteins | **Coiled-Coil** | **KLP64D/68D-S** | | |
| --- | --- | --- | --- | --- |
| Temperature (K) | 300 | 278 | 300 | 373 |
| Protein Atoms | 3346 | 3389 | 3389 | 3389 |
| Counterions(Na^+^) | 24 | 17 | 17 | 17 |
| Water | 298629 | 313122 | 313122 | 313122 |
| Total Atoms | 301999 | 316528 | 316528 | 316528 |
| Box Size (nm^3^) | 25.6**x**8.2**x**12.3 | 25.6**x**9.2**x**12.3 | 25.6**x**9.2**x**12.3 | 25.6**x**9.2**x**12.3 |
