# Supplementary material for: Biochemical and Molecular Dynamic Simulation Analysis of a Weak Coiled Coil Association between Kinesin-II Stalks
Source: PLoS One. 2012 Sep 28;7(9):e45981. doi: 10.1371/journal.pone.0045981 (PMC3461054; doi:10.1371/journal.pone.0045981)
Supplement: Table S4 — List of Salt-Bridges in KLP64D/68D-S after 20 ns at 300 K. (DOC) [file pone.0045981.s012.doc]

**Table S4. List of Salt-Bridges in KLP64D/68D-S after 20 ns at 300K:**

| **Index** | |  |  | **ATOM1** |  | | | --- | --- | --- | --- | --- | | **Res Seq** | **Res Name** | **Chain ID** | **Atom No** | **Atom Name** | | | | | | |  |  | **ATOM2** |  | | | --- | --- | --- | --- | --- | | **Res Seq** | **Res Name** | **Chain ID** | **Atom No** | **Atom Name** | | | | | | **Distance** |
| --- | --- | --- | --- | --- | --- | --- | --- | --- | --- | --- | --- | --- | --- | --- | --- | --- | --- | --- | --- | --- | --- | --- | --- | --- | --- | --- | --- | --- | --- | --- | --- |
| 1 | 435 | GLU | 64D | 119 | OE1 | 432 | LYS | 68D | 1771 | NZ | 3.46153 |
| 2 | 436 | LYS | 64D | 130 | NZ | 431 | ASP | 68D | 1760 | OD1 | 3.40952 |
| 3 | 436 | LYS | 64D | 130 | NZ | 431 | ASP | 68D | 1761 | OD2 | 3.73142 |
| 4 | 436 | LYS | 64D | 130 | NZ | 435 | GLU | 68D | 1804 | OE1 | 3.87481 |
| 5 | 436 | LYS | 64D | 130 | NZ | 435 | GLU | 68D | 1805 | OE2 | 3.48574 |
| 6 | 443 | LYS | 64D | 208 | NZ | 435 | GLU | 68D | 1804 | OE1 | 2.93143 |
| 7 | 443 | LYS | 64D | 208 | NZ | 435 | GLU | 68D | 1805 | OE2 | 3.94518 |
| 8 | 503 | GLU | 64D | 803 | OE1 | 495 | LYS | 68D | 2407 | NZ | 3.36113 |
| 9 | 503 | GLU | 64D | 804 | OE2 | 495 | LYS | 68D | 2407 | NZ | 3.06282 |
| 10 | 513 | LYS | 64D | 906 | NZ | 510 | GLU | 68D | 2573 | OE1 | 3.04475 |
| 11 | 517 | ARG | 64D | 949 | NH2 | 506 | GLU | 68D | 2533 | OE2 | 3.80098 |
| 12 | 562 | GLU | 64D | 1410 | OE1 | 559 | ARG | 68D | 3098 | NH1 | 3.89696 |
| 13 | 576 | GLU | 64D | 1564 | OE1 | 573 | LYS | 68D | 3239 | NZ | 3.20038 |
| 14 | 576 | GLU | 64D | 1565 | OE2 | 573 | LYS | 68D | 3239 | NZ | 3.10077 |
